# Burden of type 1 and type 2 diabetes and high fasting plasma glucose in Europe, 1990-2019: a comprehensive analysis from the global burden of disease study 2019

**DOI:** 10.3389/fendo.2023.1307432

**Published:** 2023-12-13

**Authors:** Dong Liang, Xiuli Cai, Qing Guan, Yangjiang Ou, Xiaoxin Zheng, Xiuquan Lin

**Affiliations:** ^1^ The School of Health Management, Fujian Medical University, Fuzhou, Fujian, China; ^2^ The School of Public Health, Fujian Medical University, Fuzhou, Fujian, China; ^3^ “The 14th Five-Year Plan” Application Characteristic Discipline of Hunan Province (Clinical Medicine), Hunan Provincial Key Laboratory of the Traditional Chinese Medicine Agricultural Biogenomics, Changsha Medical University, Changsha, Hunan, China; ^4^ Department of Cardiology, Renmin Hospital of Wuhan University, Wuhan, Hubei, China; ^5^ Cardiovascular Research Institute, Wuhan University, Wuhan, Hubei, China; ^6^ Hubei Key Laboratory of Cardiology, Wuhan, Hubei, China; ^7^ Department for Chronic and Noncommunicable Disease Control and Prevention, Fujian Provincial Center for Disease Control and Prevention, Fuzhou, Fujian, China

**Keywords:** Europe burden of disease, type 1 and type2 diabetes, chronic noncommunicable diseases, population attributable fraction, high fasting plasma glucose, risk factors

## Abstract

**Introduction:**

With population aging rampant globally, Europe faces unique challenges and achievements in chronic disease prevention. Despite this, comprehensive studies examining the diabetes burden remain absent. We investigated the burden of type 1 and type 2 diabetes, alongside high fasting plasma glucose (HFPG), in Europe from 1990-2019, to provide evidence for global diabetes strategies.

**Methods:**

Disease burden estimates due to type 1 and type 2 diabetes and HFPG were extracted from the GBD 2019 across Eastern, Central, and Western Europe. We analyzed trends from 1990 to 2019 by Joinpoint regression, examined correlations between diabetes burden and Socio-demographic indices (SDI), healthcare access quality (HAQ), and prevalence using linear regression models. The Population Attributable Fraction (PAF) was used to described diabetes risks.

**Results:**

In Europe, diabetes accounted for 596 age-standardized disability-adjusted life years (DALYs) per 100,000 people in 2019, lower than globally. The disease burden from type 1 and type 2 diabetes was markedly higher in males and escalated with increasing age. Most DALYs were due to type 2 diabetes, showing regional inconsistency, highest in Central Europe. From 1990-2019, age-standardized DALYs attributable to type 2 diabetes rose faster in Eastern and Central Europe, slower in Western Europe. HFPG led to 2794 crude DALYs per 100,000 people in 2019. Type 1 and type 2 diabetes burdens correlated positively with diabetes prevalence and negatively with SDI and HAQ. High BMI (PAF 60.1%) and dietary risks (PAF 34.6%) were significant risk factors.

**Conclusion:**

Europe’s diabetes burden was lower than the global average, but substantial from type 2 diabetes, reflecting regional heterogeneity. Altered DALYs composition suggested increased YLDs. Addressing the heavy burden of high fasting plasma glucose and the increasing burden of both types diabetes necessitate region-specific interventions to reduce type 2 diabetes risk, improve healthcare systems, and offer cost-effective care.

## 1 Introduction

Chronic non-communicable diseases (NCDs) were a leading cause of death worldwide. The United Nations (UN) and the World Health Organization (WHO) prioritized the prevention of chronic non-communicable diseases, focusing on five major disease groups: cardiovascular diseases, cancers, chronic obstructive pulmonary diseases, diabetes, and mental health (1). It was estimated that in 2019 there were 463 million people with diabetes globally, with about 15.4% in Europe. The prevalence of diabetes was projected to increase by 50% by 2045 (2). From 1990 to 2019, the burden of diabetes had continually risen, imposing substantial healthcare and economic burdens globally (3). Type 2 diabetes patients represented the majority of people with diabetes, while type 1 diabetes accounted for only 5-10%. Both types of diabetes presented varying degrees of disease burden. Moreover, high fasting plasma glucose (HFPG), one of the diagnostic criteria for diabetes, impacts the disease burden of other diseases, particularly chronic non-communicable diseases (4).

Due to early industrialization and urbanization, most regions and countries in Europe were economically advanced. However, significant differences existed between Eastern, Central, and Western Europe in terms of geography, culture, population, and diabetes management strategies (5). Europe entered an aging demographic earlier, bearing a heavier risk of disease burden associated with chronic diseases. Currently, no related studies focus on the disease burden of diabetes in Europe. A comprehensive analysis of the overall disease burden of diabetes in Europe will not only provide a basis for preventing and controlling the disease burden of diabetes in Europe but will also be of significant reference value to areas globally where the disease burden of diabetes is high.

Our study aimed to utilize the Global Burden of Diseases Study 2019 (GBD 2019) data to describe the disease burden of type 1 and type 2 diabetes and high fasting plasma glucose in various regions, timelines, and populations across Europe. Additionally, it sought to explore the associated factors to fill this knowledge gap and provide valuable insights for global diabetes prevention and control.

## 2 Materials and methods

### 2.1 Data sources

GBD 2019 systematically assessed the epidemiological characteristics of 369 diseases and injuries, and 87 risk factors in 204 countries and territories from 1990 to 2019 (6, 7). GBD 2019 provided mortality, prevalence, incidence, years of life lost (YLLs), Years lived with disability (YLDs), and disability-adjusted life years (DALYs), segmented by genders, ages, periods, geographies, and causes. This study leveraged data from GBD 2019 to analyze the burden of diabetes in 44 European countries over the same period. All data were extracted from the GBD results tool (https://vizhub.healthdata.org/gbd-results/). Additionally, the Healthcare Access and Quality (HAQ) Index and the Socio-demographic Index (SDI) can be accessed from the GBD 2019 data resource homepage (https://ghdx.healthdata.org/gbd-2019).

### 2.2 Countries of European regions

GBD 2019 classified Europe into Eastern, Central, and Western Europe. Eastern Europe included Belarus, Estonia, Latvia, Lithuania, Moldova, Russia, and Ukraine. Central Europe included Albania, Bosnia and Herzegovina, Bulgaria, Croatia, Czech Republic, Hungary, Montenegro, North Macedonia, Poland, Romania, Serbia, Slovakia, and Slovenia. Western Europe included Andorra, Austria, Belgium, Cyprus, Denmark, Finland, France, Germany, Greece, Iceland, Ireland, Israel, Italy, Luxembourg, Malta, Monaco, Netherlands, Norway, Portugal, San Marino, Spain, Sweden, Switzerland, and the UK.

### 2.3 Disease hierarchies and risk factors

We investigated non-communicable diseases at the secondary level and their associated subgroups. In GBD 2019 diseases and injuries were classified into 4 hierarchies. Level 1 included infectious diseases, maternal diseases, neonatal diseases, nutritional diseases, non-communicable diseases like diabetes, cancer, cardiovascular diseases, neurological disorders, sensory organ diseases; and injuries. Level 2 refined to level 1 into 22 disease groups: Diabetes and Chronic Kidney Disease (CKD), cardiovascular diseases; neoplasms (e.g., colon, liver, breast, ovary, pancreas, lung), sense organ diseases, neurological disorders; and tuberculosis. Level 3 detailed diabetes and CKD categories. Level 4 was subdivided into type 1 diabetes and type 2 diabetes.

We investigated risk factors at the secondary level. In GBD 2019 risk factors were classified into 4 hierarchies Level 1 included environmental/occupational factors, behavioral factors, and metabolic factors. Level 2 comprised 19 risk groups, including air pollution, inappropriate temperature, tobacco usage, dietary factors, physical inactivity, high fasting plasma glucose, and high BMI.

### 2.4 Data extraction

We extracted estimates of the death rates, DALYs, YLDs, and YLLs associated with diabetes in Eastern, Central, and Western Europe from 1990 to 2019 in GBD 2019. YLL is calculated by multiplying deaths by the remaining life expectancy. YLD derives from multiplying disease prevalence by the respective disability weight, adjusted for complications. DALYs is the sum of YLLs and YLDs. We also extracted the Population Attributable Fractions (PAFs) of type 1 and type 2 diabetes burden due to each of its risk factors. The SDI indicates developmental status, encompassing per capita income, total fertility rate (age <25 years), and average educational attainment (for those age ≥15 years), with values between 0-1 (6). The HAQ index evaluates individual healthcare access and quality per country, based on risk-standardized death rates in conditions that shouldn’t lead to death when high-quality healthcare is available. The index ranges from 0-100, and higher scores indicate better healthcare accessibility and quality (8).

### 2.5 Statistical analysis

Based on the GBD 2019 database, our study utilized descriptive statistical analysis to examine the temporal, spatial, and demographic distribution of diabetes disease burden in Europe from 1990 to 2019. The Joinpoint regression model was utilized to analyze the trends in age-standardized death rates and age-standardized DALYs globally, across Europe, and specifically within Eastern, Central, and Western Europe, over the span of 1990-2019. We employed a linear regression model to assess the impact of SDI, HAQ, and diabetes prevalence on the diabetes burden. A two-sided P-value less than 0.05 was considered statistically significant. Given that the age-standardized DALYs of 44 European countries did not meet the criteria for normal distribution, they were log-transformed to fit a normal distribution. Hence, linear regression analysis was performed with SDI, HAQ, diabetes prevalence, and the logarithm (Lg) of DALYs, yielding the regression coefficient. The research was accomplished by SPSS (version 24.0), Joinpoint (version 5.0.2) and R (version 4.2.1).

## 3 Results

### 3.1 Burden of type 1 and type 2 diabetes

In 2019, both types of diabetes caused 995 (95% UI 780-1240) crude DALYs per 100,000 people across Europe, with type 2 diabetes accounting for 93.2%. The burden of both types of diabetes was notably higher in males than females in Europe, and it escalated with age increment. The age-standardized mortality rate and DALYs rate for type 1 diabetes in Europe in 2019 were 0.6 per 100,000 people and 53.8 per 100,000 people respectively, decreasing by 40.1% (AAPC -1.74) and 2.4% compared to 1990. From 1990 to 2019, the global age-standardized death rate for type 2 diabetes increased by 10.8% (AAPC 0.36), whereas it decreased by 18.8% (AAPC -0.72) in Europe. However, during the same period, the age-standardized DALYs rate in Europe increased by 18.2% (AAPC 0.58), which was consistent with the global trend.

**Figure 1:**
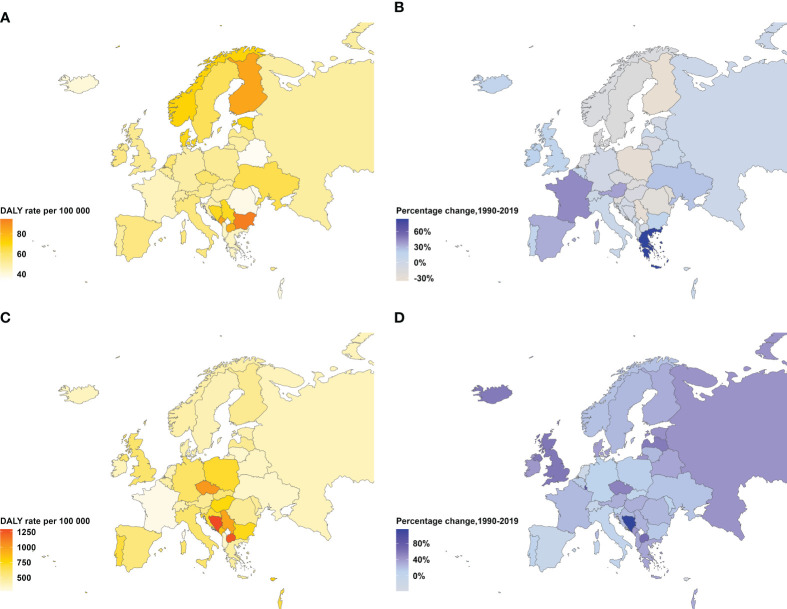
Age-standardized DALYs rate in 2019 and percentage change in DALYs rate from 1990–2019 for type 1 and type 2 diabetes, both sexes. **(A)** Age-standardized type 2 diabetes DALYs rate per 100,000 people in 2019. **(B)** Percentage change in age-standardized type 2 diabetes DALYs rate, 1990–2019. **(C)** Age-standardized type 1 diabetes DALYs rate per 100,000 adults aged 20 years or older in 2019. **(D)** Percentage change in age-standardized type 1 diabetes DALYs rate, 1990–2019. DALYs=disability-adjusted life-years.

From 1990 to 2019, compared to Western Europe (12.2%, AAPC 0.94), the age-standardized DALYs of diabetes grew more rapidly in Eastern Europe (29.6%, AAPC 0.63) and Central Europe (21.2%, AAPC 0.37). During this period, the age-standardized mortality rate of type 1 diabetes in Eastern Europe demonstrated an oscillatory trend, reaching its apex in 1994, which signified a notable inflection point of change, followed by a trend towards reduction. Concurrently, Central Europe reported a decline of 42.2% (AAPC -1.89) in age-standardized death rate for type 1 diabetes, with a more precipitous decrease noted from 1990 to 2014. On the contrary, in Eastern Europe, age-standardized mortality rates of Type 2 diabetes presented an undulating increase, amplifying by 48.3% (AAPC 0.46). Particular periods such as 1990-1994 and 2010-2016 witnessed significant rises with APCs at 6.11 and 9.69 respectively, while a pronounced upward trajectory was observed in Central Europe between 2001 and 2007 (APC 1.76). From 1990 to 2019, Western Europe recorded substantial reductions in age-standardized mortality rates for both type 1 and type 2 diabetes, showing decreases of 50.1% (AAPC -2.39) and 34.6% (AAPC -1.49) respectively, with a rapid decline from 1990 to 2014 followed by a gradual state of increment. As of 2019, Central Europe topped European ranks for age-standardized DALYs rates for both type 1 and type 2 diabetes, standing at 54.4 per 100,000 and 730.2 per 100,000 respectively. The countries in Central Europe with the heaviest burden of type 1 diabetes were Bulgaria and Montenegro, while Bosnia and Herzegovina and North Macedonia, also in Central Europe, bore the greatest burden of type 2 diabetes. However, compared to the 1990 levels, Central Europe saw a reduction of 18.8% (AAPC -0.72) in age-standardized DALYs rates for type 1 Diabetes, highlighted by a swift decline during 1997-2000 (APC -2.77) (**Tables 1**, **2**; **Table S2**; **Figures S2-5**, **Figure 1**).

**Table 1:** Age-standardized rates of deaths and DALYs due to type 1 and type 2 diabetes in Europe in 1990 and 2019, and percentage changes from 1990 to 2019.

	Age-standardized death rate per 100000			Age-standardized DALYs rate per 100000		
	1990	2019	Percentage change 1990-2019	1990	2019	Percentage change 1990-2019
T1DM						
Global	1.2 (1.0,1.4)	1.0 (0.9,1.2)	-20.4% (-31.4,-2.7)	62.3 (51.3,71.7)	57.4 (49.1,67.2)	-7.8% (-18.5,5.5)
Europe	1.0 (0.8,1.1)	0.6 (0.5,0.7)	-40.1% (-47.1,-27.2)	55.1 (46.8,64.6)	53.8 (41.8,69.4)	-2.4% (-14.8,10.2)
Eastern Europe	0.7 (0.6,0.9)	0.6 (0.5,0.9)	-16.0% (-28.6,7.0)	49.1 (42.1,57.8)	52.3 (42.2,64.8)	6.6% (-3.5,17.0)
Central Europe	1.6 (1,3,1.9)	0.9 (0.8,1.2)	-42.2% (-52.0,-28.9)	66.9 (76.2,58.0)	54.4 (44.3,67.3)	-18.8% (-30.0,-6.9)
Western Europe	0.8 (0.6,0.9)	0.4 (0.3,0.5)	-50.1% (-55.3,-37.4)	48.0 (58.6,39.1)	52.5 (38.1,71.5)	9.3% (-6.3,26.0)
T2DM						
Global	16.7 (15.7,17.5)	18.5 (17.2,19.7)	10.8% (4.4,17.4)	628.3 (537.2,730.9)	801.5 (670.6,954.4)	27.6% (22.0,33.0)
Europe	11.8 (11.0,12.2)	9.6 (8.7,10.2)	-18.8% (-22.7,-14.4)	459.2 (380.3,551.2)	542.6 (419.1,680.7)	18.2% (9.7,24.8)
Eastern Europe	4.1 (3.9,4.3)	6.1 (5.4,6.8)	48.3% (32.3,65.5)	281.5 (221.9,347.8)	376.0 (295.1,468.2)	33.6% (28.1,39.2)
Central Europe	11.7 (11.2,12.2)	11.9 (10.4,13.6)	1.7% (-10.7,14.7)	580.4 (470.4,703.7)	730.2 (559.0,923.1)	25.8% (17.0,32.8)
Western Europe	13.1 (12.1,13.6)	8.5 (7.6,9.1)	-34.6% (-37.8,-32.0)	458.4 (381.2,551.6)	515.8 (389.5,663.9)	12.5% (1.8,21.3)

**Table 2:** The annual average percent change of age-standardized rates of deaths and DALYs due to type 1 and type 2 diabetes in Europe from 1990 to 2019.

	Age-standardized death rate			Age-standardized DALYs rate		
	AAPC (95%CI)	T value	P value	AAPC (95%CI)	T value	P value
T1DM						
Global	-0.76 (-0.83, -0.7)	-22.069	<0.001	-0.27 (-0.33, -0.22)	-9.571	<0.001
Europe	-1.74 (-1.92, -1.56)	-18.754	<0.001	-0.07 (-0.27, 0.14)	-0.635	0.525
Eastern Europe	-0.4 (-1.53, 0.74)	-0.693	0.488	0.29 (-0.2, 0.79)	1.163	0.244
Central Europe	-1.89 (-2.07, -1.7)	-19.864	<0.001	-0.72 (-0.84, -0.6)	-11.696	<0.001
Western Europe	-2.39 (-2.6, -2.18)	-22.036	<0.001	0.3 (0.19, 0.4)	5.605	<0.001
T2DM						
Global	0.36 (0.32, 0.41)	16.033	<0.001	0.82 (0.73, 0.9)	19.63	<0.001
Europe	-0.72 (-0.91, -0.53)	-7.408	<0.001	0.58 (0.45, 0.7)	9.21	<0.001
Eastern Europe	1.46 (0.68, 2.25)	3.683	<0.001	1.04 (0.82, 1.26)	9.477	<0.001
Central Europe	0.07 (-0.08, 0.22)	0.869	0.385	0.77 (0.67, 0.86)	15.908	<0.001
Western Europe	-1.49 (-1.58, -1.41)	-33.79	<0.001	0.39 (0.3, 0.48)	8.675	<0.001

**Figure 2:**
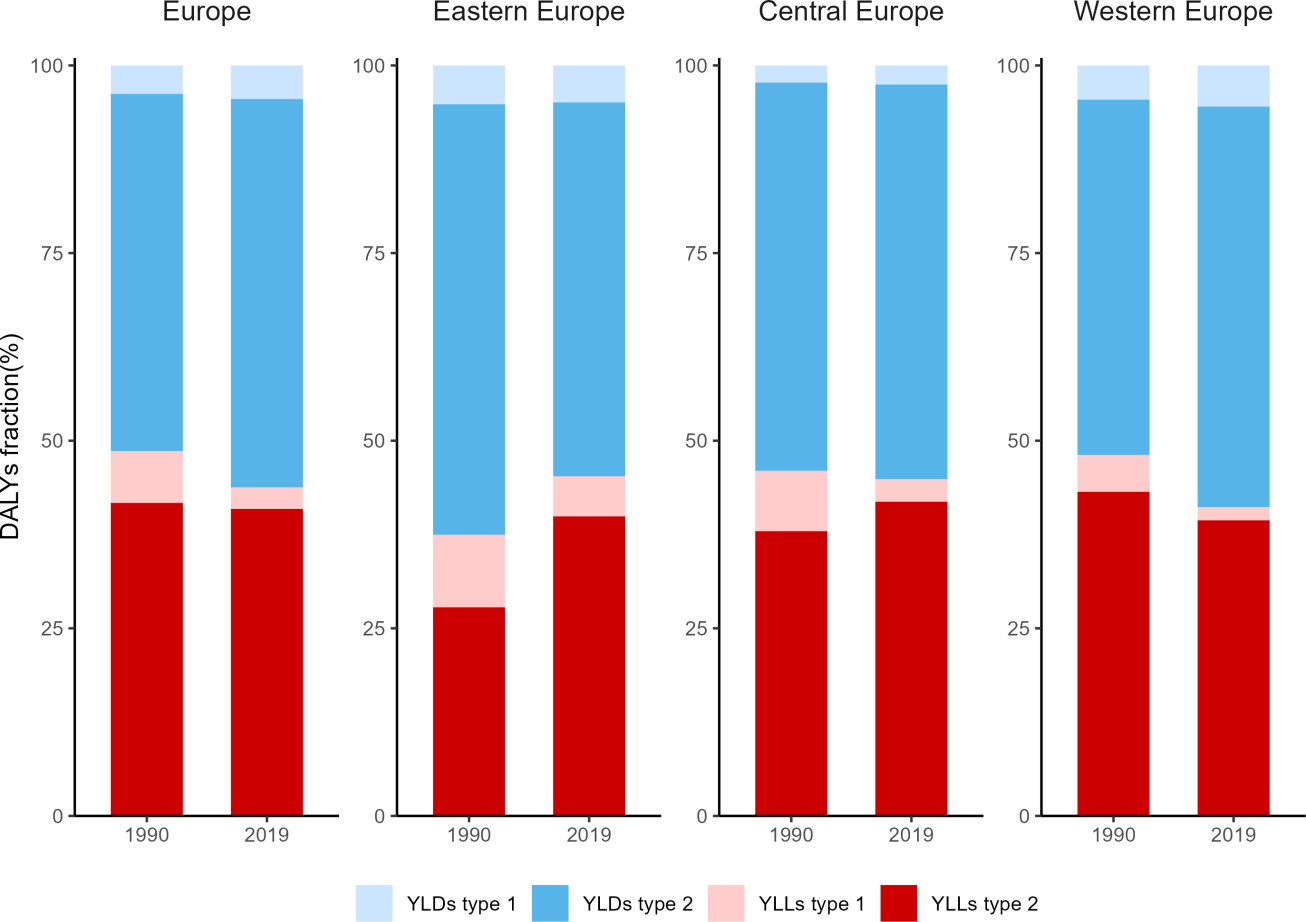
Fractions of YLLs and YLDs for type 1 and type 2 diabetes in 1990 and 2019. YLLs, Years of life lost; YLDs, years lived with disability.

In 1990, YLLs and YLDs were nearly equally represented in Europe's diabetes burden. However, by 2019, this burden shifted towards YLDs as Central and Western Europe regions reported an increased YLD proportion in DALYs over the period 1990-2019, while the YLLs fraction decreased. Eastern, Central, and Western Europe all registered YLDs ratios above 50%, peaking at 58.8% in Western Europe. For type 1 diabetes in 1990, the European YLLs rate exceeded the YLDs rate, which was contrastingly lower for type 2 diabetes. In 2019, the YLLs proportion in type 1 diabetes receded below the YLDs proportion. Furthermore,Eastern, Central and Western Europe saw an approximate 50% distribution between YLLs and YLDs (**Figure 2**).

### 3.2 Burden of high fasting plasma glucose

**Figure 3:**
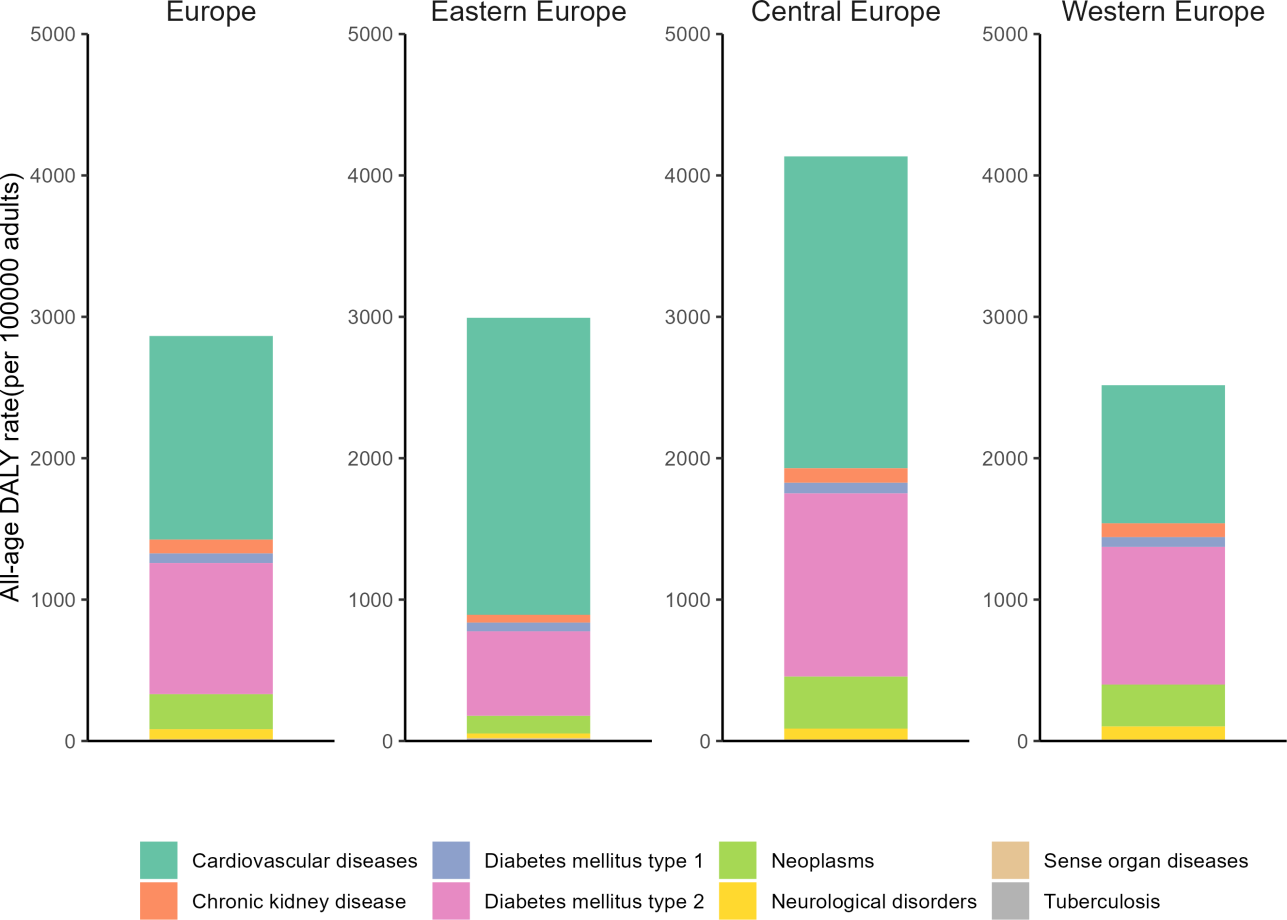
All-age DALYs rate due to high fasting plasma glucose in Europe by cause in 2019.

In 2019, HFPG resulted in 2864 crude DALYs per 100,000 people in Europe, with 35.6% of this burden originating from type 1 and type 2 diabetes. As outlined in the GBD 2019, the significant impact of HFPG predominantly manifested in non-communicable chronic diseases, such as cardiovascular diseases, chronic kidney disease, neoplasms, neurological disorders, tuberculosis, and other sensory disorders. Significantly, cardiovascular diseases attributable to HFPG constituted a remarkable 50.3% of the overall burden in Europe. Amongst all regions, Central Europe bore the maximum burden of HFPG, while considerable regional variations were observed in the cardiovascular disease burden attributable to HFPG, with Eastern and Central Europe notably surpassing Western Europe. Specifically, Central Europe and Eastern Europe attributed 53.3% and an alarming 70.2% of their cardiovascular disease burden to HFPG, respectively (**Table S4**; **Figure 3**).

### 3.3 Determinants: SDI, HAQ, and diabetes prevalence

**Figure 4:**
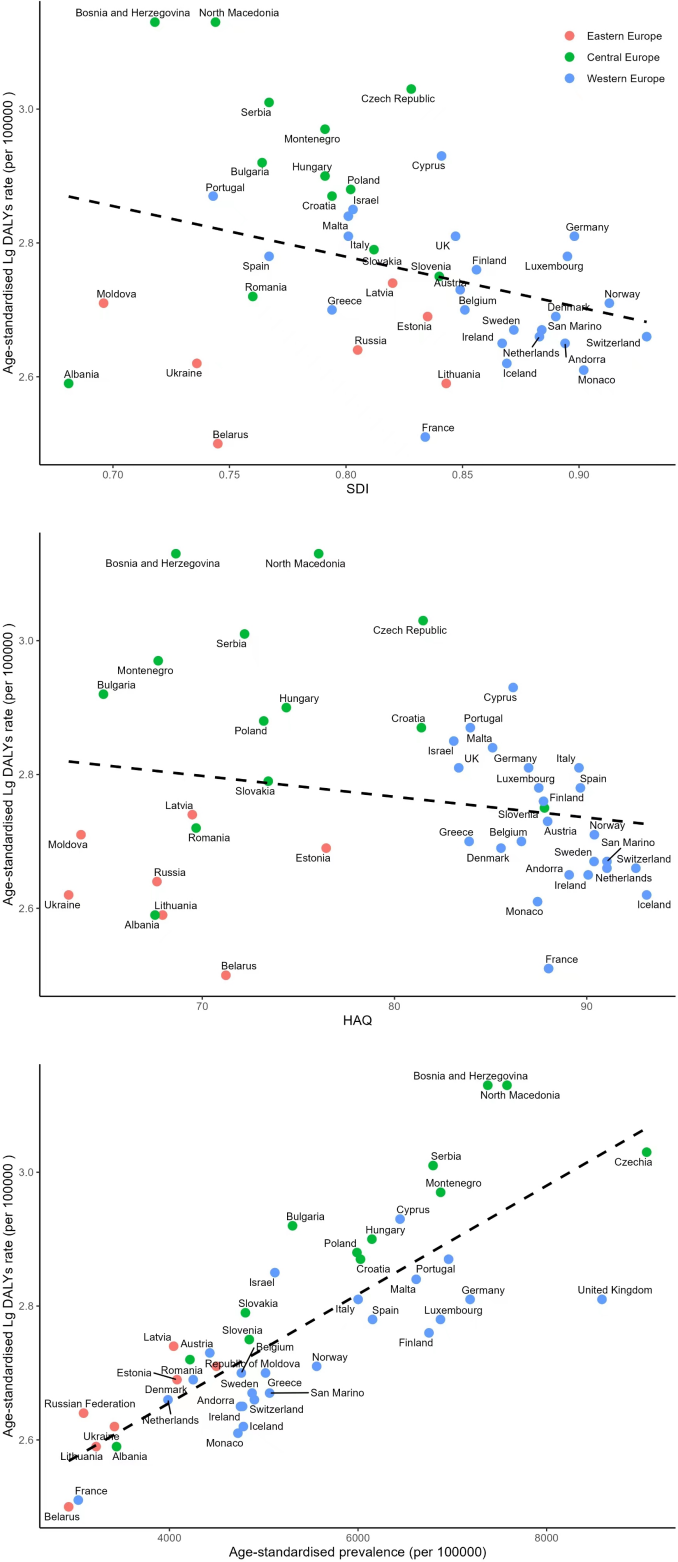
Association of the Socio-demographic Index (SDI), the Healthcare Access and Quality Index (HAQ) and prevalence with DALYs due to diabetes.

Linear regression analysis revealed that the logarithm of age-standardized DALYss (Lg DALYss) was associated with SDI and diabetes prevalence. There was a negative correlation between SDI and Lg DALYs (R2 = 0.096, P=0.037). Higher SDI values indicated lower diabetes burdens, as evidenced in Western Europe countries with high SDI and relatively low diabetes burden. There’s no significant correlation between HAQ and Lg DALYs (R2 = 0.006, P=0.611). However, after excluding data from Eastern Europe countries that deviated from the regression line, a negative correlation between the HAQ index and Lg DALYs emerged (R2 = 0.310, P<0.001). Diabetes prevalence correlated positively with Lg DALYs (R2 = 0.659, P<0.001). Higher diabetes prevalence leads to greater diabetes disease burdens (**Figure 4**).

Given the strong positive correlation between European diabetes prevalence and Lg DALYss, we explored the prevalence and incidence of type 1 and type 2 diabetes. In 2019, type 1 diabetes prevalence exceeded the global average, while type 2 diabetes prevalence was below it. Regional differences were observed in the rates of both types. Western Europe showed the highest standardized prevalence for type 1 diabetes and the most significant growth (69.3%). Central Europe exhibited the highest age-standardized prevalence for type 2 diabetes. Increases in prevalence were paralleled with the rise in incidence rates for both Type 1 (69.1%) and Type 2 (47.4%) diabetes (**Table S3**).

### 3.4 Risk factors

**Figure 5:**
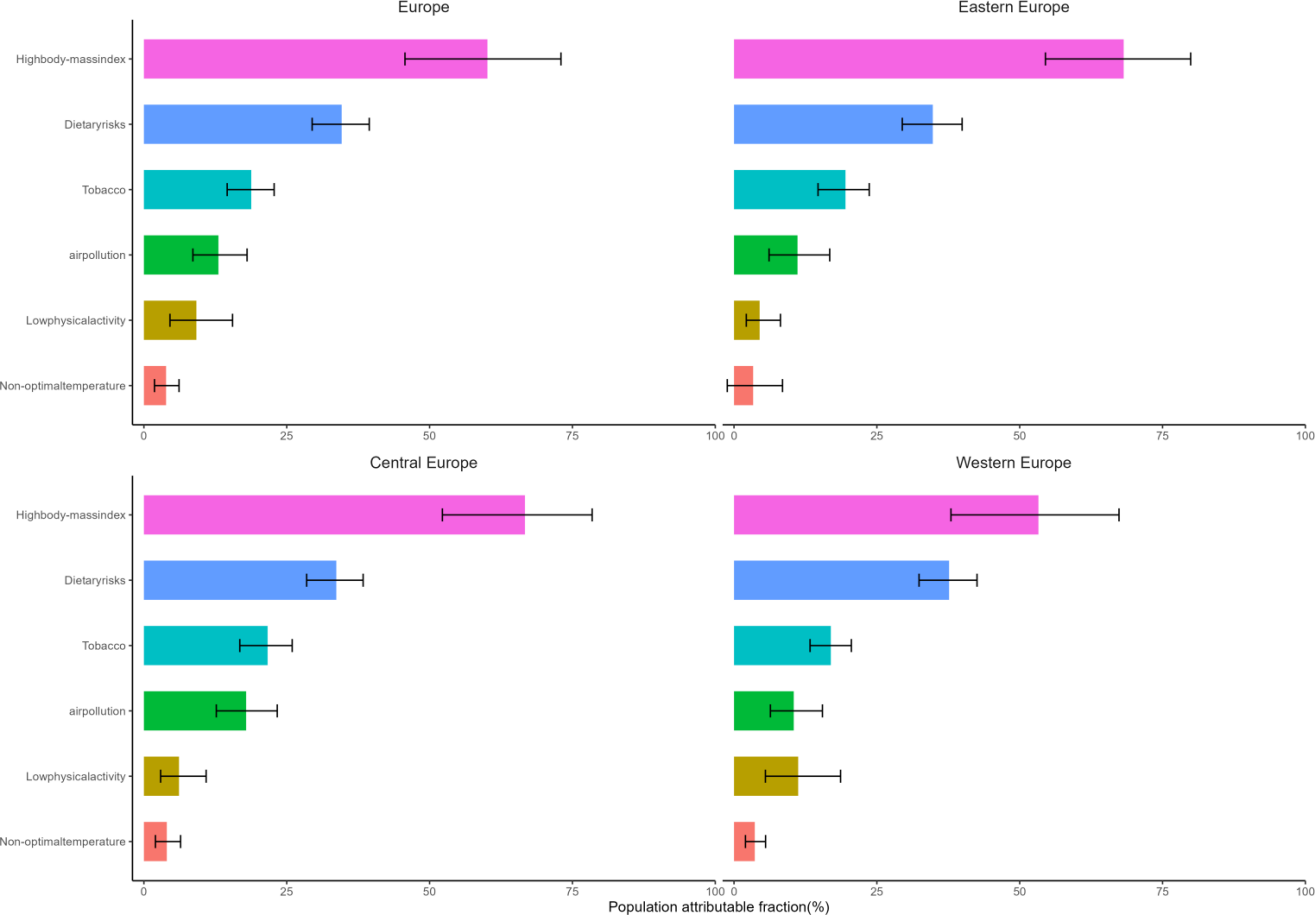
Population attributable fraction of crude DALYs due to type 2 diabetes for the main risk factors identified by GBD 2019, by region.

In 2019, high body mass index (BMI) emerged as the leading risk factor for the burden of type 2 diabetes in Europe. The influence of this factor varied across regions, ranging from 53.3% in Western Europe to 68.2% in Eastern Europe. Dietary risks accounted for 34.6% of DALYs, including excessive consumption of red and processed meats, sugary beverages, insufficient whole grains, fruits, dietary fibers, seeds, and nuts. Tobacco and air pollution emerged as the third and fourth leading contributors, respectively. Across different European regions, the prevalent risk factors, in descending order, were high BMI, diet, tobacco, air pollution, low physical activity, and non-optimal temperatures. Of particular note was the fact that Western Europe had the highest proportion of diabetes burden attributable to dietary factors and physical inactivity, yet the lowest linked to high BMI. In contrast, Eastern Europe recorded the highest percentage of disease burden associated with air pollution (**Figure 5**).

## 4 Discussion

From 1990 to 2019, both the age-standardized rates of death and DALYs for type 1 and type 2 diabetes in Europe were below global levels. This was contrary to expectations given the significant aging population in Europe (9), which should theoretically bear a severe burden of diabetes. This fact indicated that Europe had achieved commendable results in managing diabetes against the backdrop of an aging population, possibly due to economic growth and developments in healthcare. Compared to type 1 diabetes, type 2 diabetes contributed a heavier burden to the total DALYs from diabetes (93.2%). Therefore, future diabetes prevention and control strategies in Europe should still lean towards type 2 diabetes.

The burden of diabetes varied across different regions in Europe. From 1990 to 2019, the age-standardized death rates for type 1 diabetes have significantly decreased in Eastern, Central, and Western Europe. While the exact reasons for this decrease were unclear, insulin supplements (10), genetics (11), and environmental risk factors (12) played crucial roles in enhancing life expectancy. However, age-standardized death rates for type 2 diabetes showed regional variations: they were growing in Eastern and Central Europe while decreasing by 34.6% in Western Europe. The decline in mortality rates from type 1 and type 2 diabetes in Western Europe, along with the prevalence of acute and chronic complications (13, 14), had led to an increase in the proportion of YLDs component within total DALYs, far exceeding the YLLs component. age-standardized DALYs rates for type 1 diabetes increased by 6.6% and 9.3% in Eastern and Western Europe respectively, while decreasing by 18.8% in Central Europe. At the same time, age-standardized DALYs rates for type 2 diabetes continued to grow in all three regions of Europe, albeit at a slower pace in Western Europe.

The study results showed that SDI and HAQ were significant factors affecting the burden of diabetes, and they explained the variations in the distribution and changes in the burden of diabetes across different regions of Europe. Firstly, the 2019 SDI and HAQ data for Eastern, Central, and Western Europe demonstrated that the levels of economic development and healthcare in Eastern and Central Europe lag behind those in Western Europe, resulting in heavier burdens of diabetes. Compared to Western European countries (13), Eastern and Central European countries lacked the corresponding infrastructure and capabilities, suffered from inadequate discretionary health expenditure (15, 16) and public resources (17, 18), hence preventing them from introducing advanced diabetes treatment technologies such as the Hybrid Closed-Loop (HCL) system. A key issue with adopting novel medical technologies was the shortage of funds to purchase new medical equipment. Economic constraints also affected the level of education accessible by physicians and diabetic patients, hindering their understanding and absorption of innovative medical technologies, and impacting the establishment of professional diabetes care in medical institutions. Therefore, the main task to alleviate the growing burden of diabetes, which increased as diabetes prevalence rose, was to tackle public health issues in relatively underdeveloped countries in Eastern and Central Europe. Secondly, looking at the changes in SDI from 1990 to 2019 in Eastern, Central, and Western Europe, Western Europe had entered a plateau phase, while Eastern and Central Europe maintained rapid development. Specifically, Central Europe was progressively bridging the economic disparity with Western Europe, spurred by its accelerated growth. In the foreseeable future, it is likely to emerge as a subsequent parallel to Western Europe. However, to avoid falling into the same predicament of diabetes burden as Western Europe, Central Europe can learn from the situation in Western Europe and adjust their diabetes management strategies as needed.

During the period of 1990 to 2019, the mortality rate trend associated with type 2 diabetes in Europe was in contrast to the global pattern, while the age-standardized Disability Adjusted Life Years (DALYs) continued on an upward trajectory. This signifies that apart from the aging demographic, additional risk components contribute substantially to the burden of type 2 diabetes. Given that type 2 diabetes contributed considerably to the overall burden of diabetic diseases, and the risk factors for type 2 diabetes also served as significant prognostic factors for type 1 diabetes, research had demonstrated the feasibility and effectiveness of prevention and treatment programs for type 2 diabetes (19, 20). Therefore, primary prevention of diabetes is of great importance. The risk factors for type 2 diabetes discussed in our study included high BMI, dietary factors, tobacco use, lack of exercise, and air pollution. High BMI has been proven to be one of the major factors in the development of diabetes (21), serving as an essential indicator for measuring overweight and obesity. In recent decades, almost all European countries have seen an increase in overweight/obesity rates (22). If no intervention measures were taken, it is expected to escalate further (23). Lifestyle improvements could help prevent high-risk individuals (24). Existing research evidence suggested that a healthy lifestyle was the best measure for preventing and managing diabetes (25). Consequently, implementing health education targeting individuals with diabetes could serve as a crucial strategy for diabetes management and prevention, fostering a shift in health perception leading to self-initiated healthy activities. We should adhere to healthy eating habits, mirroring models like the Mediterranean diet and Nordic dietary patterns (26), and control our energy intake while focusing on nutritional balance to ensure sufficient nutrient intake. It was important to participate in physical exercise actively and maintain a stable frequency and intensity of workouts, thereby enhancing physical vitality and metabolic capacity. We could maintain a stable and healthy weight through diet and exercise, reducing the likelihood of overweight and obesity. In recent years, numerous studies had demonstrated that air pollution could lead to insulin resistance (5), while particulate matter and persistent organic pollutants could increase the risk of diabetes (27, 28) and obesity (29). As such, air pollution has emerged as one of the risk factors for diabetes. Therefore, it is necessary to restrict the emission of polluting gases through more stringent legislation, strictly enforce air pollutant emission standards, intensify penalties, and improve related environmental engineering projects to mitigate the harm of air pollution to human health. Additionally, efforts to promote smoking cessation through education should be increased, along with further refinement of laws and regulations related to smoking bans in public places.

HFPG was considered the primary risk factor for CNDs. It could exert influences not only on diabetes as well other various diseases, acting as a pivotal risk factor contributing to the increased global and regional disease burden (30). Compared to the burden of diabetes alone, the overall burden of HFPG was heavier, effectively doubling in magnitude. As indicated by data from Europe, diabetes and cardiovascular diseases were the primary causes of health loss in the region. A cohort study based on biobank data testified that prediabetes and type 2 diabetes were linked with cardiovascular diseases, chronic kidney disease, and heart failure (31). Furthermore, high glycemia-induced oxidative stress may cause cardiovascular damage, establishing a relationship between hyperglycemia and an increased risk of cardiovascular diseases (32). Additionally, there existed an inextricable link between cardiovascular diseases (CVDs) and diabetes; CVDs were the leading cause of morbidity and mortality in patients with both type 1 and type 2 diabetes (33, 34). The onset of cardiovascular disease as a complication of diabetes resulted in a doubled mortality rate and reduced life expectancy by at least 12 years (35). Consequently, in order to alleviate the disease burden in the European region, emphasis must be put on managing these two conditions. Furthermore, given that HFPG was a risk factor for numerous diseases, efforts should be made to mitigate this risk, while also considering the impact of low FPG values.

To sum up, there existed substantial heterogeneity in the burden of diabetes across Europe: 1) Variation in diabetes type. Type 2 diabetes continued to constitute the crux of future prevention and control strategies in Europe; 2) Geographical discrepancies. Central Europe stood at the forefront of diabetes burden within Europe; 3) Differentials in the composition of diabetes burden. Owing to the falling mortality rate from diabetes and the high prevalence of acute and chronic complications, YLDs accounted for a significant share of the diabetes burden in Western Europe. Despite Europe’s onset of ageing dating back to the 19th century, its age-standardized death rate from diabetes and DALYs remained below global averages. Although the rise in diabetes burden has markedly decelerated in Western Europe - the most economically advanced region of Europe, the proportion of YLDs was escalating swiftly. This serves as both a caution and model for other rapidly developing European regions and countries worldwide that are witnessing an ageing demographic. To counteract the burden of diabetes and HFPG levels, it is imperative to initiate primary healthcare interventions targeting the diabetic population with the aim of mitigating diabetes risk, whilst simultaneously ensuring efficient management of diabetes-associated complications, notably cardiovascular diseases.

Our study has its limitations, some of which are inherent to the GBD study (6). Firstly, certain European countries lack accurate, high-quality data; therefore, it becomes necessary to rely on data from other countries that have been adjusted for covariates. However, this inevitably introduces bias into the obtained results, a situation frequently observed during the evaluation process for type 1 diabetes and non-fatal burden. Secondly, Within the context of the GBD, we lean towards using ample data sources and embracing the effects brought by adjustments, rather than exclusively relying on limited high-quality data sources. Although utilizing other case definitions for diabetes as adjustment strategies can garner more data sources for analysis, it also signifies introducing measurement bias. Finally, there are seven countries in Eastern Europe, a small number, of which resulted in no correlation between the diabetes disease burden in Europe and the Healthcare Access and Quality (HAQ) Index. However, when data from Eastern Europe was excluded and linear regression analysis was applied solely to Central and Western European countries, a strong correlation existed between the HAQ Index and diabetes burden. The HAQ Index, within the GBD, served as a covariate for estimating the fatal and non-fatal burden of diabetes in countries with sparse data, hence the association between the index and the diabetes burden may be overstated.

## Data availability statement

The original contributions presented in the study are included in the article/**Supplementary Material**. Further inquiries can be directed to the corresponding authors.

## Author contributions

DL: Writing – original draft, Writing – review & editing. XC: Writing – original draft. Writing – review & editing. QG: Writing – review & editing. YO: Writing – review & editing. XZ: Writing – review & editing. XL: Writing – review & editing.
