# Supplementary material for: Burden of type 1 and type 2 diabetes and high fasting plasma glucose in Europe, 1990-2019: a comprehensive analysis from the global burden of disease study 2019
Source: Front Endocrinol (Lausanne). 2023 Dec 13;14:1307432. doi: 10.3389/fendo.2023.1307432 (PMC10752242; doi:10.3389/fendo.2023.1307432)
Supplement: Supplementary file 1 [file DataSheet_1.docx]

Supplementary Material

# Supplementary Figures and Tables

## **Supplementary Figures**

## Figure S1 Age distribution of DLAYs rate due to type 1 and type 2 diabets across Europe in 2019


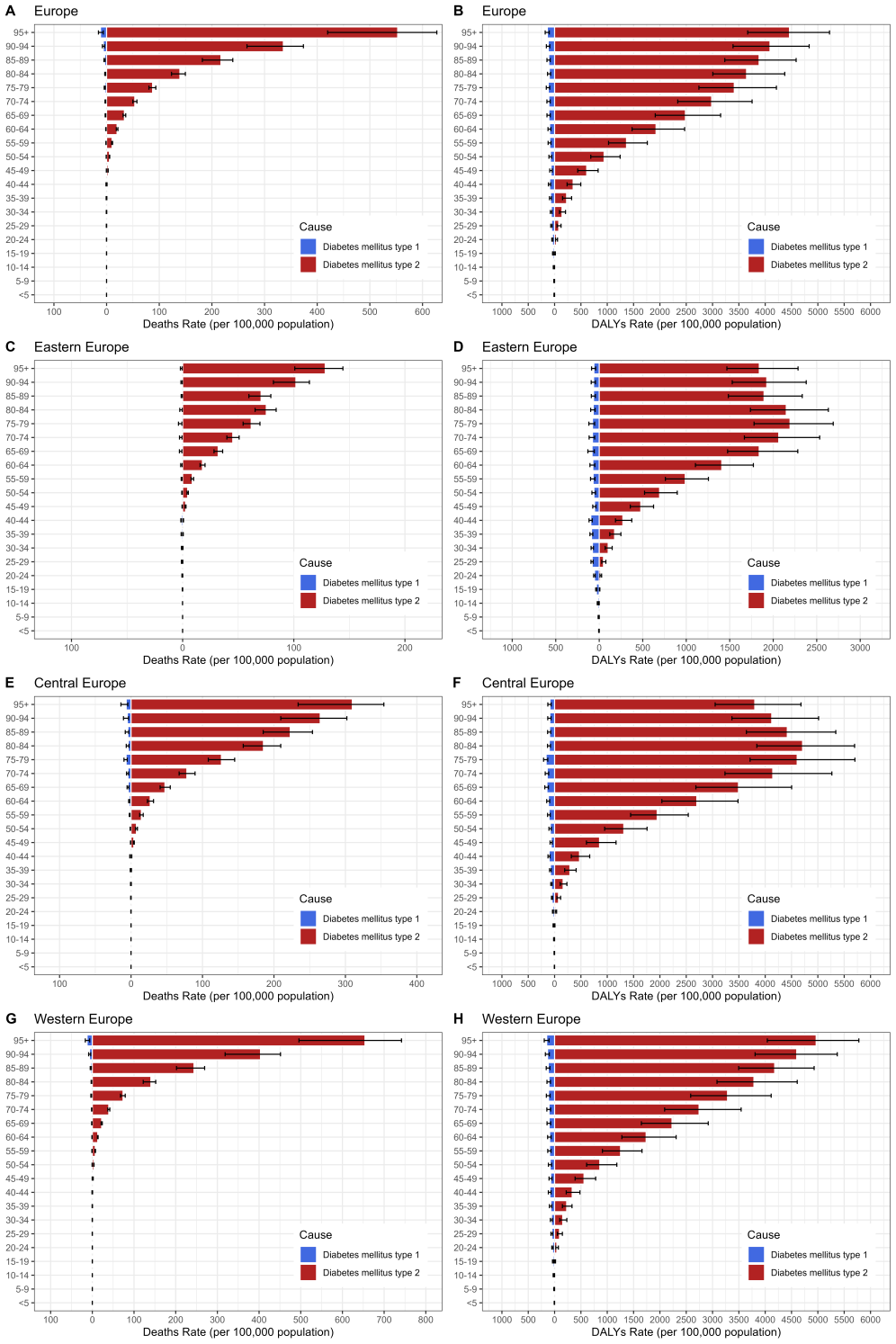


## Figure S2 Joinpoint regression analysis of Age-standardized DALYs of type 1 and type 2 diabetes at Global, Europe, Eastern Europe, Centarl Europe and Western Europe levels from 1990 to 2019.


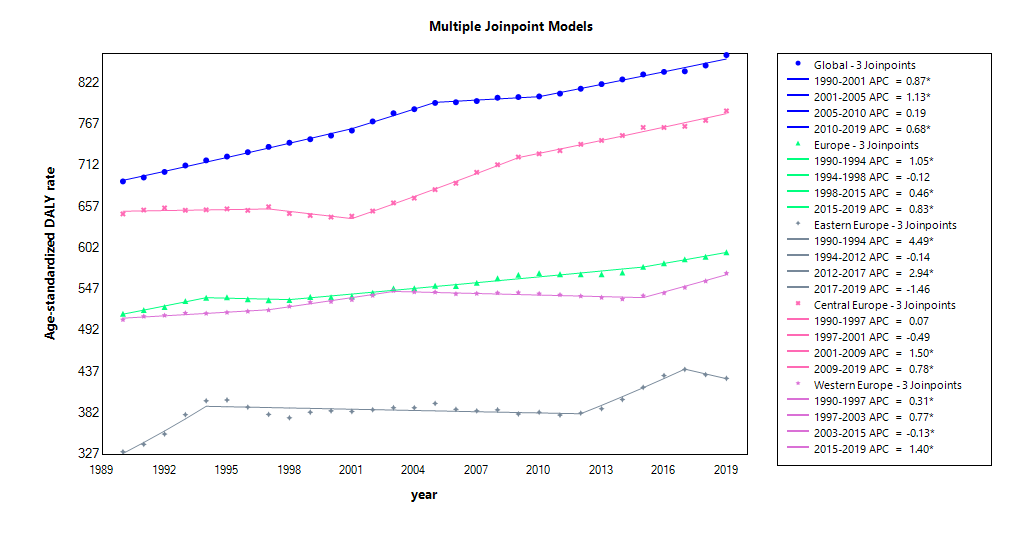


## Figure S3 Joinpoint regression analysis of Age-standardized death rates of type 1 diabetes in Global, Europe, Eastern Europe, Centarl Europe and Western Europe levels from 1990 to 2019.


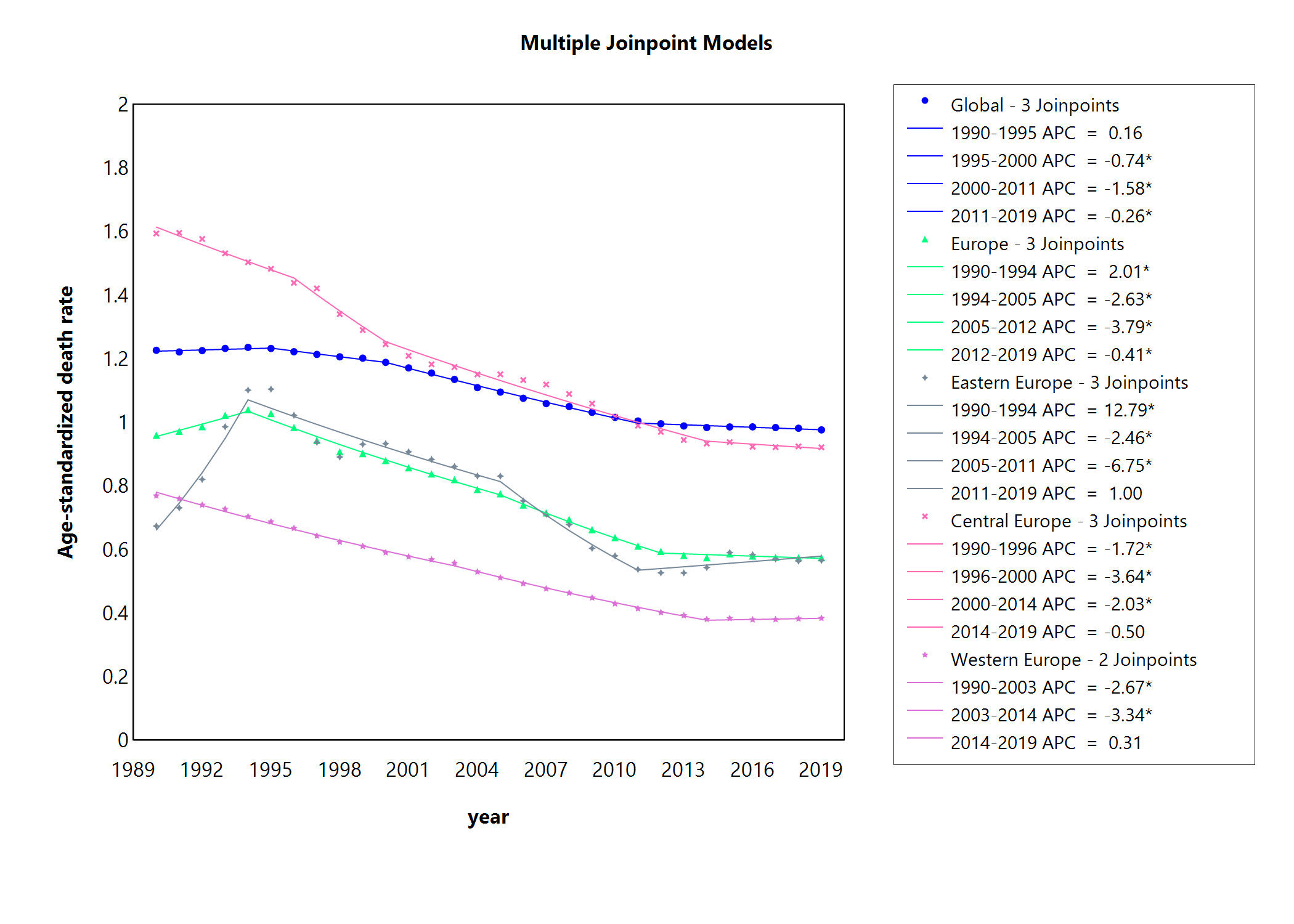


## Figure S4 Joinpoint regression analysis of Age-standardized death rates of type 2 diabetes in Global, Europe, Eastern Europe, Centarl Europe and Western Europe levels from 1990 to 2019.


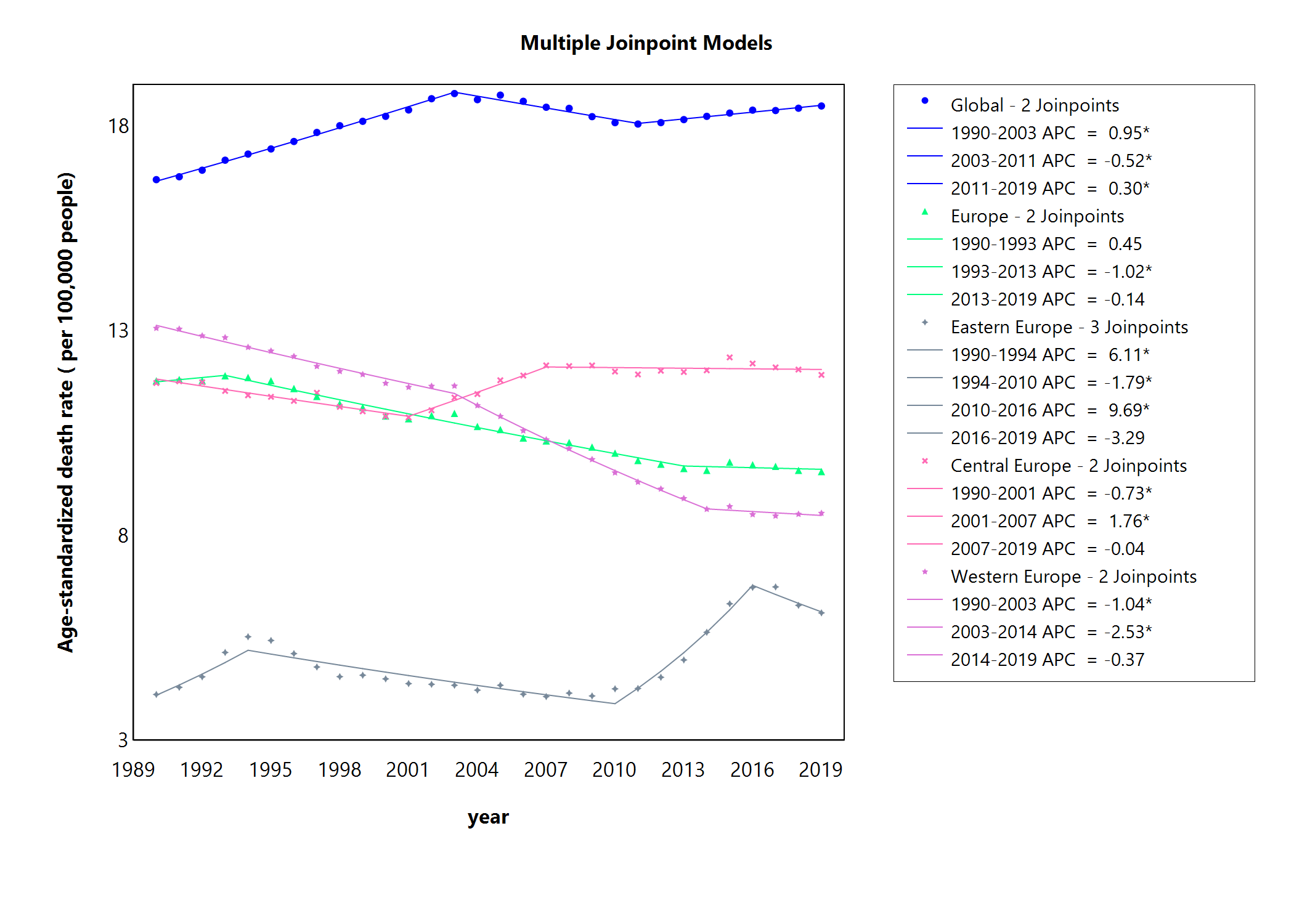


## Figure S5 Joinpoint regression analysis of Age-standardized DALYs of type 1 diabetes in Global, Europe, Eastern Europe, Centarl Europe and Western Europe levels from 1990 to 2019.


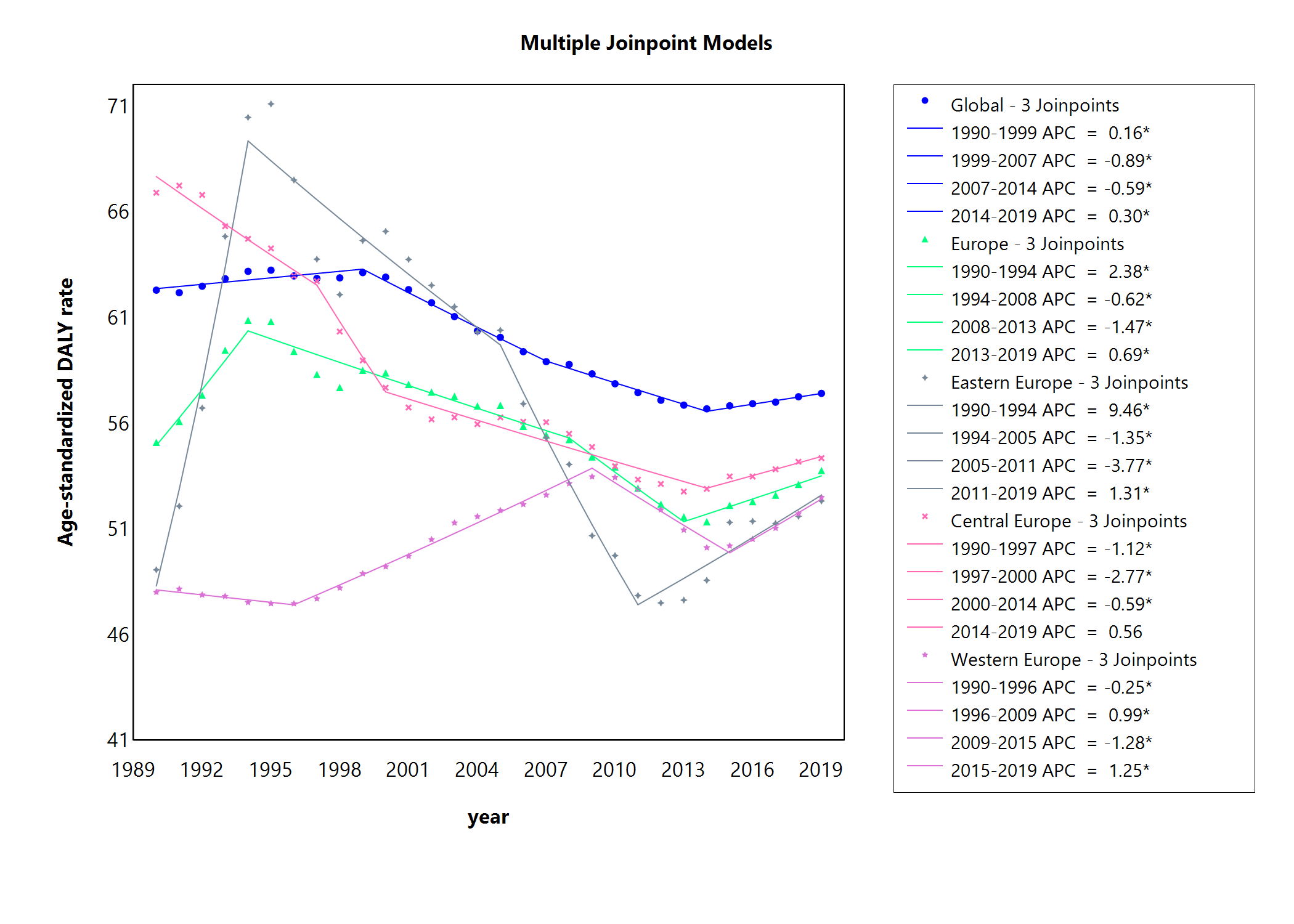


## Figure S6 Joinpoint regression analysis of Age-standardized DALYs of type 2 diabetes in Global, Europe, Eastern Europe, Centarl Europe and Western Europe levels from 1990 to 2019.


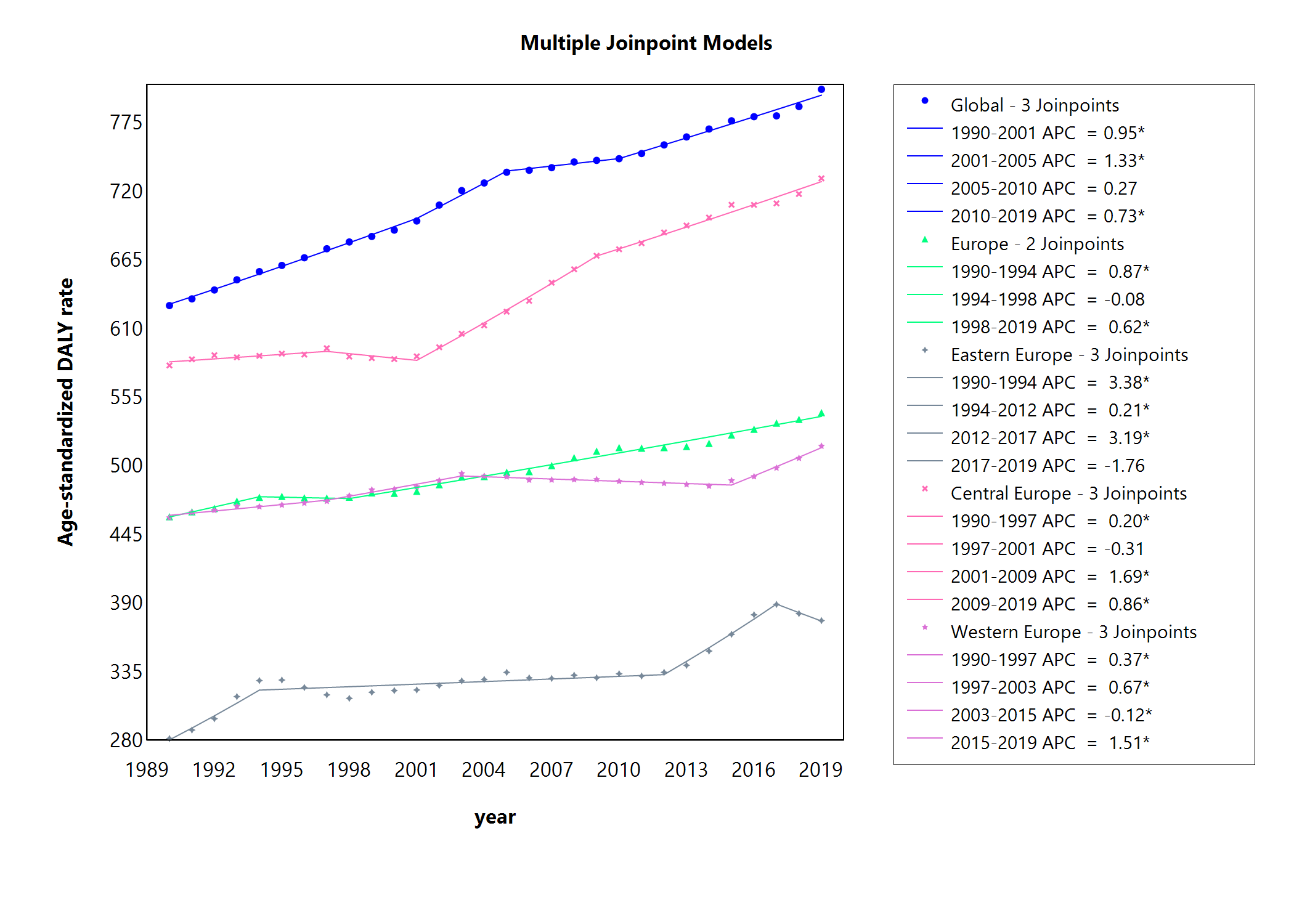


## **1.2 Supplementary Tables**

Table S1 List of regions and countries of the Europe

| Location | Countries of Europe |
| --- | --- |
| Eastern Europe | Republic of Moldova; Ukraine; Latvia; Russian Federation; Belarus; Lithuania; Estonia |
| Central Europe | Albania, Bosnia and Herzegovina, Bulgaria, Croatia, Czech Republic, Hungary, Montenegro, North Macedonia, Poland, Romania, Serbia, Slovakia, and Slovenia |
| Western Europe | Andorra, Austria, Belgium, Cyprus, Denmark, Finland, France, Germany, Greece, Iceland, Ireland, Israel, Italy, Luxembourg, Malta, Monaco, Netherlands, Norway, Portugal, San Marino, Spain, Sweden, Switzerland, and the UK |

## Table S2 All-age DALYs, age-standardised DALYs due to type 1 and type 2 diabetes in 2019 and percentage change from 1990-2019, percentage of type 2 diabetes

| Location | All-ages DALYs 2019 | | Age-standardised DALYs | |
| --- | --- | --- | --- | --- |
|  | Rate(per 100000) | Percentage type 2 diabetes  %(95% UI) | rate(per 100000) | Percentage change  1990-2019 |
| Global | 916.1(771,7,1087.9) | 93.5(92.9,93.9) | 859.0(723.5,1019.9) | 24.4%(18.5%,29.7%) |
| Europe | 995.0(780.2,1240.1) | 93.2(92.7,93.2) | 596.3(463.9,744.3) | 16.0%(7.5%,22.9%) |
| Eastern Europe | 658.6(524.1,811.9) | 90.7(89.9,91.6) | 428.3(340.8,527.3) | 29.6%(24.2%,35.2%) |
| Central Europe | 1370.0(1068.9,1715.1) | 94.5(93.9,94.7) | 784.6(608.0,985.2) | 21.2%(11.7%,28.4%) |
| Western Europe | 1042.6(804.5,1330.1) | 93.4(93.1,93.7) | 568.2(430.1,733.1) | 12.2%(1.6%,20.9%) |

## Table S3 The prevalence and incidence of type 1 and type 2 diabetes in 1990 and 2019 and their change from 1990 to 2019

|  | Prevalence (age-standardised rate) | | | Incidence (age-standardised rate) | | |
| --- | --- | --- | --- | --- | --- | --- |
|  | 1990 | 2019 | % change 1990-2019 | 1990 | 2019 | % change1990-2019 |
| T1DM | | | | | | |
| Global | 211.8(171.6,257.6) | 272.5(217.0,337.0) | 28.7%(24.3,33.5) | 5.6(4.6,6.8) | 7.6(6.2,9.3) | 36.3%(31.9,41.3) |
| Europe | 289.3(240.1,344.7) | 483.9(385.3,600.0) | 67.3%(57.3,77.3) | 8.0(6.8,9.5) | 13.6(11.0,16.6) | 69.1%(59.7,78.3) |
| Eastern Europe | 229.6(183.3,286.0) | 337.2(266.5,419.6) | 46.8%(43.9,49.8) | 7.0(5.7,8.7) | 10.0(8.1,12.4) | 42.3%(39.3,44.8) |
| Central Europe | 197.8(159.9,242.3) | 333.2(262.3,416.5) | 68.5%(60.7,77.7) | 5.6(4.7,6.8) | 9.4(7.6,11.6) | 66.3%(59.0,75.0) |
| Western Europe | 366.1(309.9,427.0) | 619.9(490.5,764.9) | 69.3%(55.2,84.7) | 10.2(8.7,11.8) | 17.4(14.1,21.1) | 70.9%(57.7,85.6) |
| T2DM | | | | | | |
| Global | 3546.5(3862.7,3243.7) | 5282.9(4853.6,5752.1) | 49.0%(47.1,50.6) | 184.6(170.9,199.7) | 259.9(240.3,281.4) | 40.8%(39.3,42.4) |
| Europe | 3090.8(2828.2,3358.4) | 4789.1(4337.6,5249.6) | 55.0%(51.1,58.9) | 165.5(153.5,178.2) | 244.0(224.0,264.1) | 47.4%(44.0,50.8) |
| Eastern Europe | 2167.6(1966.9,2399.9) | 2856.6(2582.5,3157.0) | 31.8%(28.1,35.6) | 105.4(96.7,114.9) | 142.6(131.0,156.0) | 35.3%(32.3,38.9) |
| Central Europe | 3663.7(3984.4,3342.5) | 5619.9(5099.5,6110.2) | 53.4%(49.7,57.3) | 191.9(178.0,207.2) | 286.0(263.2,309.9) | 49.0%(45.5,52.7) |
| Western Europe | 3339.3(3028.1,3637.9) | 5360.8(4812.9,5915.5) | 60.5%(54.3,67.3) | 187.9(173.2,202.5) | 276.4(252.2,301.2) | 47.1%(41.8,52.7) |

## Table S4 The sex specific death count and age-standardized deaths rate of type 1 and type 2 diabetes in 2019

|  | Death count | | Age-standardized deaths rate per 100000 | |
| --- | --- | --- | --- | --- |
|  | Male | Female | Male | Female |
| T1DM | | | | |
| Global | 40521.01  (35481.02, 51281.12) | 37715.11  (29953.43, 47461.23) | 18.21 (16.48, 19.66) | 20.98 (19.53, 22.53) |
| Europe | 3368.40  (3181.57, 5667.85) | 2648.13  (2647.15, 4907.76) | 9.23 (8.17, 9.96) | 11.22 (10.42, 11.93) |
| Eastern Europe | 749.02  (606.96, 1130.47) | 653.16  (468.98, 1425.22) | 6.9 (5.95, 7.98) | 6.03 (5.22, 6.91) |
| Central Europe | 967.55  (744.82, 1434.9) | 765.11  (600.36, 1138.97) | 11.3 (9.74, 12.91) | 14.54 (12.62, 16.65) |
| Western Europe | 1650.85  (1175.99, 2613.87) | 1228.86  (817.49, 1852.41) | 7.62 (6.59, 8.22) | 10.38 (9.66, 10.92) |
| T2DM | | | | |
| Global | 714568.44  (666622.09, 763932.16) | 758365.54  (684910.51, 819621.06) | 19.94 (18.5, 21.32) | 17.3 (15.62, 18.7) |
| Europe | 60913.52  (64424.62, 74062.9) | 80919.61  (80714.09, 90714.55) | 10.51 (9.71, 11.2) | 8.78 (7.76, 9.49) |
| Eastern Europe | 6632.98  (5680.28, 7707.43) | 14922.3  (12733.63, 17210.54) | 5.38 (4.62, 6.2) | 6.43 (5.47, 7.43) |
| Central Europe | 11998.36  (10382.26, 13801.64) | 14840.22  (12650.78, 16924.19) | 13.37 (11.6, 15.33) | 10.61 (9.05, 12.13) |
| Western Europe | 42281.18  (38944.18, 44635.8) | 51156.99  (42832.06, 55972.69) | 9.87 (9.09, 10.41) | 7.37 (6.36, 7.96) |

## Table S5 The sex specific age-standardized DALYs rate of type 1 and type 2 diabetes in 2019

|  | Age-standardized DALYs rate per 100000 | |
| --- | --- | --- |
|  | Male | Female |
| T1DM |  |  |
| Global | 61.47 (52.54, 72.98) | 53.36 (43.81, 64.3) |
| Europe | 60.1 (47.48, 76.39) | 47.67 (35.92, 62.77) |
| Eastern Europe | 55.81 (45.49, 69) | 48.7 (38.02, 63.04) |
| Central Europe | 64.47 (52.27, 80.25) | 44.44 (34.58, 57.11) |
| Western Europe | 59.13 (43.79, 78.96) | 45.97 (32.56, 64.38) |
| T2DM | | |
| Global | 865.16 (721.2, 1030.7) | 743.71 (621.81, 888.99) |
| Europe | 602.58 (467.88, 750.96) | 490.69 (377.82, 618.6) |
| Eastern Europe | 372.29 (291.99, 468.52) | 374.52 (293.27, 466.94) |
| Central Europe | 833.94 (642.36, 1052.17) | 636.43 (485.73, 811.02) |
| Western Europe | 587.36 (450.02, 750.28) | 449.33 (332.64, 581.38) |

## Table S6 The age-standardized deaths rate and age-standardized DALYs rate due to type 1 diabetes. The Europe, regions, and countries

|  | Age-standardized deaths rate per 100000 | | | Age-standardized DALY rate per 100000 | | |
| --- | --- | --- | --- | --- | --- | --- |
|  | 1990 | 2019 | percentage change 1990-2019 (%) | 1990 | 2019 | percentage change 1990-2019(%) |
| Global | 1.23 (1.43, 0.97) | 0.98 (1.17, 0.85) | -20.4 (-31.4, -2.7) | 62.3 (71.67, 51.35) | 57.41 (67.23, 49.11) | -7.84 (-18.54, 5.5) |
| Europe | 0.96 (1.15, 0.78) | 0.57 (0.75, 0.51) | -40.12 (-47.14, -27.2) | 55.1 (64.59, 46.82) | 53.76 (69.36, 41.77) | -2.42 (-14.77, 10.24) |
| Eastern Europe | 0.67 (0.87, 0.6) | 0.57 (0.87, 0.47) | -16.02 (-28.58, 7) | 49.07 (57.8, 42.11) | 52.32 (64.79, 42.17) | 6.63 (-3.48, 16.98) |
| Belarus | 0.68 (0.86, 0.59) | 0.31 (0.41, 0.23) | -54.47 (-66.99, -38.94) | 46.25 (54.88, 40.06) | 35.95 (47.13, 27.08) | -22.26 (-37.45, -6.93) |
| Estonia | 1.29 (1.46, 0.95) | 0.99 (1.32, 0.75) | -23.27 (-44.59, 9.27) | 71.94 (81.18, 60.27) | 69.4 (88.05, 55.3) | -3.53 (-22.53, 18.15) |
| Latvia | 0.77 (0.86, 0.54) | 0.62 (0.81, 0.49) | -19.26 (-39.75, 18.29) | 50.56 (58.06, 40.23) | 53.58 (66.83, 43.25) | 5.97 (-13.43, 31.51) |
| Lithuania | 0.99 (1.1, 0.77) | 0.61 (0.87, 0.46) | -38.6 (-54.58, -8.16) | 56.28 (63.04, 48.65) | 49.95 (63.31, 39.76) | -11.23 (-27.57, 12.24) |
| Republic of Moldova | 0.7 (0.84, 0.58) | 0.32 (0.46, 0.25) | -54.76 (-65.14, -38.97) | 48.63 (56.39, 40.99) | 37.12 (47.93, 27.98) | -23.66 (-36.64, -9.86) |
| Russian Federation | 0.64 (0.87, 0.55) | 0.51 (0.89, 0.39) | -19.33 (-35.31, 12.71) | 48.47 (58.28, 41.1) | 50.34 (63.91, 39.84) | 3.85 (-6.8, 15.52) |
| Ukraine | 0.74 (0.9, 0.65) | 0.8 (0.99, 0.66) | 8.1 (-12.57, 33.33) | 50.72 (58.64, 44.13) | 63.35 (77.13, 51.78) | 24.89 (8.19, 43.82) |
| Central Europe | 1.59 (1.88, 1.3) | 0.92 (1.21, 0.75) | -42.18 (-52, -28.93) | 66.91 (76.25, 57.96) | 54.35 (67.27, 44.33) | -18.77 (-30.03, -6.93) |
| Albania | 0.99 (1.17, 0.86) | 0.66 (0.93, 0.47) | -33.19 (-53.83, -4.66) | 47.88 (56.56, 40.58) | 44.2 (56.96, 33.83) | -7.69 (-24.24, 10.13) |
| Bosnia and Herzegovina | 2.18 (2.49, 1.88) | 1.5 (1.93, 1.15) | -31.4 (-49.27, -9.22) | 73.08 (82.71, 65.22) | 68.18 (84.75, 54.66) | -6.7 (-24.89, 13.85) |
| Bulgaria | 1.92 (2.85, 1.66) | 2.21 (2.92, 1.42) | 14.69 (-29.45, 62.56) | 82.07 (105.77, 71.53) | 94.71 (120.05, 71.75) | 15.41 (-16, 47.58) |
| Croatia | 1.13 (1.35, 0.73) | 0.64 (0.86, 0.45) | -43.74 (-58.92, -21.83) | 49.29 (58.8, 36.77) | 47.06 (61.72, 35.29) | -4.54 (-23.35, 18.35) |
| Czechia | 1.81 (2.4, 1.39) | 1.09 (1.91, 0.79) | -39.82 (-56.27, -9.72) | 68.13 (83.54, 57.85) | 59.28 (78.35, 45.69) | -12.99 (-27.98, 4.58) |
| Hungary | 1.14 (1.52, 0.92) | 0.64 (1.14, 0.46) | -43.7 (-58.48, -15.68) | 54.93 (66.3, 46.79) | 48.89 (64.29, 36.94) | -11 (-25.65, 6.52) |
| North Macedonia | 2.45 (2.83, 2.11) | 2.05 (2.64, 1.58) | -16.25 (-37.81, 9.74) | 85.51 (97.9, 74.79) | 81.56 (101.59, 65.34) | -4.62 (-23.02, 16.43) |
| Montenegro | 2.07 (2.64, 1.74) | 1.93 (2.58, 1.53) | -6.45 (-26.42, 19.44) | 83.34 (98.7, 71.94) | 84.84 (103.59, 69.77) | 1.81 (-13.1, 20.27) |
| Poland | 1.83 (2.14, 1.31) | 0.85 (1.2, 0.67) | -53.29 (-63.27, -35.53) | 73.78 (85.38, 60.38) | 52.03 (64.86, 40.63) | -29.48 (-40.99, -17.36) |
| Romania | 0.94 (1.2, 0.75) | 0.4 (0.64, 0.3) | -57.07 (-68.04, -37.5) | 50.09 (58.46, 42.08) | 38.11 (49.79, 29.27) | -23.91 (-37.1, -7.11) |
| Serbia | 2.55 (3.01, 2.06) | 1.35 (1.72, 1.05) | -47.3 (-61.04, -27.11) | 97.03 (111.81, 81.96) | 68.26 (84.47, 54.37) | -29.66 (-43.2, -13.74) |
| Slovakia | 1.45 (1.62, 1.28) | 0.77 (1.01, 0.58) | -46.84 (-60.64, -29) | 60.71 (68.77, 53.94) | 49.44 (62.87, 38.79) | -18.56 (-34.37, -0.55) |
| Slovenia | 1.09 (1.49, 0.58) | 0.28 (0.52, 0.19) | -74.4 (-85.33, -41.41) | 42.24 (54.19, 29.72) | 33.4 (45.82, 23.67) | -20.93 (-44.3, 16.47) |
| Western Europe | 0.77 (0.93, 0.57) | 0.38 (0.52, 0.32) | -50.08 (-55.29, -37.36) | 48.01 (58.61, 39.08) | 52.49 (71.47, 38.13) | 9.34 (-6.32, 26) |
| Andorra | 0.69 (0.96, 0.51) | 0.54 (0.71, 0.4) | -22.18 (-49.31, 16.09) | 46.59 (62.44, 35.19) | 60.05 (82.55, 43.74) | 28.89 (3.38, 51.72) |
| Austria | 0.63 (0.79, 0.46) | 0.38 (0.53, 0.31) | -40.43 (-48.88, -24.36) | 39.22 (48.81, 31.25) | 53.26 (72.61, 37.9) | 35.8 (13.59, 62.07) |
| Belgium | 0.78 (0.98, 0.59) | 0.37 (0.49, 0.29) | -52.89 (-59.56, -39.53) | 47.48 (58.12, 38.4) | 51.87 (71.33, 37.03) | 9.25 (-8.44, 27.1) |
| Cyprus | 1.64 (2.26, 0.7) | 0.63 (0.81, 0.34) | -61.71 (-70.71, -45.69) | 54.63 (69.18, 36.37) | 54.39 (75.04, 38.45) | -0.43 (-24.65, 34.97) |
| Denmark | 1.65 (1.84, 1.08) | 0.93 (1.24, 0.76) | -43.9 (-52.89, -22.76) | 80.87 (94.34, 61.97) | 68.65 (87.5, 53.75) | -15.11 (-28.08, 3.69) |
| Finland | 1.92 (2.14, 1.42) | 0.66 (0.9, 0.56) | -65.81 (-72.38, -49.56) | 129.68 (155.34, 106.14) | 84.03 (109.9, 63.5) | -35.21 (-46.14, -21.71) |
| France | 0.75 (0.86, 0.45) | 0.45 (0.59, 0.35) | -39.96 (-51.53, -13.79) | 31.6 (38.16, 26.13) | 45.31 (61.94, 33.32) | 43.38 (19.89, 72) |
| Germany | 1.06 (1.25, 0.72) | 0.45 (0.65, 0.38) | -57.11 (-63.95, -39.24) | 50.68 (60.41, 39.57) | 49.67 (65.27, 37.19) | -1.99 (-20.51, 21.91) |
| Greece | 0.16 (0.23, 0.13) | 0.11 (0.16, 0.09) | -32.17 (-41.63, -20.58) | 24.13 (32.51, 17.82) | 44.44 (63.74, 29.62) | 84.15 (57.64, 115.17) |
| Iceland | 0.52 (0.61, 0.36) | 0.3 (0.45, 0.24) | -42.93 (-54.16, -15.98) | 35.71 (46.38, 27.35) | 40.6 (55.48, 29.34) | 13.71 (-1.36, 28.24) |
| Ireland | 0.72 (0.94, 0.54) | 0.33 (0.45, 0.26) | -53.67 (-60.63, -45.69) | 46.01 (58.04, 36.4) | 55.18 (76.85, 38.83) | 19.93 (0.83, 37.37) |
| Israel | 0.53 (0.7, 0.39) | 0.49 (0.58, 0.32) | -7.07 (-30.41, 8.99) | 36.21 (46.66, 27.75) | 38.87 (49.76, 29.51) | 7.35 (-6.97, 23.79) |
| Italy | 0.53 (0.95, 0.45) | 0.35 (0.49, 0.28) | -33.31 (-53.01, -22.7) | 52.57 (69.18, 39.9) | 56.12 (77.78, 39.58) | 6.75 (-7.27, 16.84) |
| Luxembourg | 0.88 (1.05, 0.63) | 0.36 (0.53, 0.29) | -59.23 (-67.51, -43.05) | 47.28 (58.14, 38.03) | 49.34 (67.71, 35.3) | 4.37 (-13.81, 24.43) |
| Malta | 1.11 (1.44, 0.82) | 0.57 (0.77, 0.47) | -48.43 (-56.97, -32.63) | 61.17 (76.21, 49.26) | 65.35 (87.42, 48.58) | 6.84 (-7.44, 20.71) |
| Netherlands | 1.56 (1.83, 1.01) | 0.56 (0.73, 0.45) | -63.86 (-69.52, -49.17) | 67.43 (80.39, 53.31) | 58.31 (78.41, 42.57) | -13.54 (-29.65, 5.18) |
| Norway | 1.03 (1.4, 0.94) | 0.59 (0.86, 0.5) | -42.91 (-49.3, -34.99) | 82.03 (100.62, 66.91) | 71.44 (94.21, 53.51) | -12.92 (-20.93, -5.36) |
| Portugal | 0.67 (0.84, 0.48) | 0.26 (0.39, 0.21) | -61.82 (-68.83, -47.17) | 44.58 (55.33, 35.74) | 50.73 (71.31, 35.29) | 13.82 (-6.01, 35.82) |
| Spain | 0.49 (0.67, 0.38) | 0.15 (0.25, 0.12) | -69.23 (-75.5, -52.85) | 41.79 (52.64, 33.02) | 55.21 (79.16, 37.01) | 32.11 (5.03, 62) |
| Sweden | 0.96 (1.14, 0.74) | 0.55 (0.73, 0.46) | -42.69 (-49.09, -31.86) | 75.24 (93.83, 59.28) | 61.84 (81.94, 45.93) | -17.8 (-25.33, -10.49) |
| Switzerland | 0.66 (0.78, 0.35) | 0.18 (0.24, 0.14) | -73.49 (-79.14, -51.71) | 49.25 (64.68, 33.76) | 49.61 (71.05, 33.95) | 0.73 (-20.47, 28.82) |
| United Kingdom | 0.54 (0.74, 0.47) | 0.34 (0.43, 0.29) | -37.56 (-43.53, -33.47) | 45.44 (57.49, 36.38) | 52.48 (72.06, 38.06) | 15.49 (1.78, 27.67) |
| Monaco | 0.54 (0.68, 0.37) | 0.35 (0.45, 0.26) | -35.54 (-54.07, -0.3) | 41.38 (54.86, 31.18) | 52.91 (74.78, 37.45) | 27.86 (9.41, 45.12) |
| San Marino | 0.6 (0.73, 0.47) | 0.42 (0.63, 0.27) | -30.41 (-56.73, 8.4) | 42.32 (55.33, 32.2) | 53.77 (74.8, 38.41) | 27.05 (6.76, 47.18) |

## Table S7 The age-standardized deaths rate and age-standardized DALYs rate due to type 2 diabetes. The Europe, regions, and countries

|  | Age-standardized deaths rate per 100000 | | | | | Age-standardized DALY rate per 100000 | | | |
| --- | --- | --- | --- | --- | --- | --- | --- | --- | --- |
|  | | 1990 | 2019 | percentage change 1990-2019 | 1990 | | 2019 | percentage change 1990-2019 |  |
| Global | | 16.69 (17.55, 15.7) | 18.49 (19.66, 17.18) | 10.77 (4.42, 17.44) | 628.33 (730.86, 537.22) | | 801.55 (954.43, 670.58) | 27.57 (21.98, 32.96) |  |
| Europe | | 11.78 (12.25, 10.95) | 9.56 (10.2, 8.67) | -18.81 (-22.7, -14.43) | 459.2 (551.23, 380.25) | | 542.57 (680.74, 419.05) | 18.16 (9.68, 24.83) |  |
| Eastern Europe | | 4.12 (4.26, 3.92) | 6.11 (6.82, 5.4) | 48.31 (32.29, 65.48) | 281.46 (347.76, 221.94) | | 375.97 (468.17, 295.07) | 33.58 (28.06, 39.19) |  |
| Belarus | | 4.21 (4.43, 3.95) | 2.21 (2.78, 1.78) | -47.48 (-58.24, -34.1) | 298.94 (379.04, 229.87) | | 278.74 (378.08, 194.9) | -6.75 (-16.83, 3.03) |  |
| Estonia | | 3.32 (3.75, 3.03) | 4.47 (5.6, 3.52) | 34.91 (3.68, 69.62) | 291.41 (379.71, 217.31) | | 421.84 (562.88, 305.48) | 44.76 (32.97, 56.59) |  |
| Latvia | | 4.61 (4.97, 4.31) | 7.69 (9.2, 6.44) | 66.87 (38.27, 100.89) | 314.66 (400.36, 242.26) | | 491.74 (637.05, 375.76) | 56.28 (42.09, 70.45) |  |
| Lithuania | | 3.52 (3.83, 3.29) | 3.71 (4.52, 3.02) | 5.29 (-14.74, 28.22) | 273.58 (352.13, 208.24) | | 339.73 (447.52, 252.03) | 24.18 (14.51, 34.19) |  |
| Republic of Moldova | | 6.57 (6.92, 6.24) | 4.61 (5.3, 3.97) | -29.82 (-39.85, -19.04) | 443.24 (557.87, 349.9) | | 478.93 (633.74, 346.4) | 8.05 (-2.5, 17.36) |  |
| Russian Federation | | 4.24 (4.4, 4.01) | 7.45 (8.47, 6.45) | 75.56 (53.21, 99.76) | 270.36 (333.19, 215.11) | | 387.91 (480.3, 307.05) | 43.48 (36.15, 51.91) |  |
| Ukraine | | 3.71 (3.88, 3.52) | 3.25 (3.78, 2.77) | -12.39 (-25.38, 2.74) | 296.92 (374.35, 231.69) | | 351.05 (451.67, 266.75) | 18.23 (8.82, 27.5) |  |
| Central Europe | | 11.73 (12.2, 11.2) | 11.92 (13.59, 10.36) | 1.68 (-10.73, 14.74) | 580.37 (703.66, 470.41) | | 730.22 (923.08, 558.96) | 25.82 (17.02, 32.84) |  |
| Albania | | 4.16 (4.61, 3.72) | 3.44 (4.55, 2.58) | -17.36 (-37.97, 9.72) | 262.47 (344.13, 200.38) | | 348.75 (469.82, 249.28) | 32.87 (20.14, 44.5) |  |
| Bosnia and Herzegovina | | 14.25 (15.56, 13.12) | 37 (45.56, 28.27) | 159.6 (81.31, 222.75) | 602.25 (728.24, 489.43) | | 1293.93 (1598.44, 1026.16) | 114.85 (84.38, 144.1) |  |
| Bulgaria | | 15.14 (16.07, 14.02) | 13.79 (16.87, 11.14) | -8.88 (-26.2, 11.96) | 638.4 (764.81, 527.55) | | 729.6 (924.46, 564.13) | 14.29 (1.16, 26.59) |  |
| Croatia | | 11.88 (12.72, 11.03) | 11.29 (13.76, 9.06) | -5.02 (-24.09, 16.16) | 566.88 (705.85, 451.94) | | 698.24 (909.46, 522.38) | 23.17 (10.79, 35.15) |  |
| Czechia | | 12.09 (12.71, 11.17) | 14.37 (17.22, 11.72) | 18.88 (-1.42, 41.67) | 666.83 (842.42, 516.54) | | 1016.48 (1325.47, 754.02) | 52.44 (39.66, 64.5) |  |
| Hungary | | 11.58 (12.12, 10.93) | 11.68 (14.06, 9.66) | 0.8 (-16.53, 20.55) | 579.72 (718.42, 461.16) | | 744.81 (961.99, 568.63) | 28.48 (17.06, 40.39) |  |
| North Macedonia | | 19.37 (21.34, 17.18) | 33.57 (40.86, 26.96) | 73.29 (34.59, 117.16) | 758.94 (918.03, 620.99) | | 1255.92 (1561.52, 994.84) | 65.48 (46.3, 86.02) |  |
| Montenegro | | 11.66 (13.13, 10.07) | 13.73 (16.38, 11.51) | 17.78 (-3.87, 47.24) | 606.22 (755.21, 471.25) | | 848.8 (1090.15, 642.28) | 40.02 (28.55, 52.31) |  |
| Poland | | 12.09 (12.69, 11.48) | 10.41 (12.2, 8.8) | -13.92 (-26.16, 0.03) | 634.83 (768.21, 520.16) | | 708.22 (902.47, 535.5) | 11.56 (1.99, 18.95) |  |
| Romania | | 6.51 (6.86, 6.18) | 5.95 (7.02, 4.87) | -8.67 (-24.33, 7.62) | 387.94 (483.92, 306.49) | | 482.75 (624.98, 364.61) | 24.44 (12.7, 35.78) |  |
| Serbia | | 18.81 (21.31, 15.58) | 21.4 (25.9, 17.62) | 13.78 (-11.64, 45.02) | 755.95 (911.01, 617.36) | | 962.84 (1206.37, 749.56) | 27.37 (12.37, 42.74) |  |
| Slovakia | | 12.63 (13.73, 11.58) | 8.2 (10.2, 6.47) | -35.02 (-49.89, -17.03) | 549.28 (667.37, 444.33) | | 561.02 (734.71, 419.48) | 2.14 (-10.34, 13.99) |  |
| Slovenia | | 10.47 (13, 8.27) | 7.06 (8.93, 5.56) | -32.57 (-51.54, -9.21) | 499.35 (633.94, 385.64) | | 524.59 (690.09, 370.8) | 5.05 (-8.98, 17.69) |  |
| Western Europe | | 13.06 (13.56, 12.12) | 8.55 (9.07, 7.64) | -34.57 (-37.82, -32.02) | 458.39 (551.61, 381.2) | | 515.75 (663.92, 389.52) | 12.52 (1.83, 21.26) |  |
| Andorra | | 7.25 (9.68, 5.52) | 5.55 (7, 4.27) | -23.51 (-46.38, 6.12) | 285.71 (367.04, 219.81) | | 387.41 (514.19, 283.86) | 35.6 (13.58, 54.76) |  |
| Austria | | 12.16 (12.76, 11.27) | 10.98 (11.88, 9.8) | -9.67 (-16.46, -2.67) | 378.8 (453.07, 319.4) | | 486.96 (623.07, 378.24) | 28.55 (16.79, 40.22) |  |
| Belgium | | 11 (11.68, 10.08) | 6.08 (6.61, 5.38) | -44.71 (-48.83, -40.48) | 398.58 (496.41, 318.01) | | 451.98 (600.27, 327.19) | 13.4 (1.54, 25.02) |  |
| Cyprus | | 63.98 (74.6, 49.74) | 26.88 (30.97, 21.94) | -57.99 (-65.34, -48.65) | 1282.68 (1499.3, 1081.34) | | 794.82 (979.27, 639.92) | -38.03 (-47.16, -28.12) |  |
| Denmark | | 9.26 (9.9, 8.7) | 10.83 (11.78, 9.76) | 16.93 (6.54, 26.99) | 303.8 (353.66, 261.23) | | 417.49 (512.55, 338.85) | 37.43 (25.25, 48.83) |  |
| Finland | | 6.44 (7.04, 5.87) | 3.34 (3.65, 2.95) | -48.07 (-53.81, -42.59) | 376.06 (478.65, 288.46) | | 489.43 (664.06, 343.8) | 30.15 (15.97, 42.12) |  |
| France | | 7.76 (8.35, 7.07) | 7.13 (7.79, 6.25) | -8.1 (-15.9, -0.01) | 225.56 (266.32, 190.72) | | 278.2 (345.74, 220.64) | 23.34 (12.7, 34.25) |  |
| Germany | | 15.37 (16.27, 14.17) | 9.65 (10.46, 8.63) | -37.2 (-41.68, -32.74) | 558.52 (685.65, 457.13) | | 602.22 (783.66, 453.18) | 7.82 (-2.98, 17.8) |  |
| Greece | | 7.5 (7.89, 6.97) | 5 (5.4, 4.54) | -33.28 (-38.48, -27.91) | 342.07 (429.19, 265.27) | | 456.55 (610.28, 329.62) | 33.47 (20.33, 45.64) |  |
| Iceland | | 4.77 (5.23, 4.3) | 3.92 (4.45, 3.36) | -17.69 (-28.22, -5.59) | 240.03 (311.19, 185.4) | | 377.78 (509.76, 273.05) | 57.39 (43.25, 70.07) |  |
| Ireland | | 9.91 (10.45, 9.28) | 6.1 (6.71, 5.33) | -38.47 (-44.48, -32.54) | 275.49 (322.85, 236.13) | | 388.72 (515.54, 284.43) | 41.1 (19.1, 61.95) |  |
| Israel | | 17.09 (18, 15.82) | 20.62 (22.3, 18.22) | 20.65 (10.95, 29.67) | 514.04 (603.96, 436.49) | | 674.77 (825.62, 549.59) | 31.27 (22.67, 38.99) |  |
| Italy | | 19.03 (19.75, 17.8) | 11.92 (12.71, 10.39) | -37.33 (-42.14, -33.88) | 583.36 (680.72, 499.42) | | 586.64 (742.84, 455.5) | 0.56 (-9.15, 9.13) |  |
| Luxembourg | | 10.17 (10.99, 9.26) | 5.79 (6.7, 4.94) | -43.09 (-50.56, -34.36) | 299.48 (356.77, 251.92) | | 556.16 (741.56, 399.42) | 85.71 (55.09, 114.96) |  |
| Malta | | 23.12 (24.89, 21.21) | 11.8 (13.36, 10.15) | -48.98 (-55.33, -41.85) | 693.36 (824.37, 584.83) | | 629.41 (805.99, 483.94) | -9.22 (-19.04, 0.09) |  |
| Netherlands | | 16.56 (17.64, 15.08) | 8.51 (9.29, 7.61) | -48.58 (-52.36, -44.2) | 494.54 (579.31, 418.96) | | 401.05 (516.58, 304.68) | -18.9 (-28.41, -7.91) |  |
| Norway | | 6.75 (7.1, 6.15) | 5.65 (6.05, 5.01) | -16.34 (-21.24, -11.38) | 370.43 (466.88, 289.98) | | 438.45 (572.08, 327.29) | 18.36 (11.45, 23.18) |  |
| Portugal | | 19.92 (20.87, 18.7) | 14.49 (15.76, 12.73) | -27.27 (-33.78, -21.13) | 654.82 (774.15, 558.36) | | 684.53 (868.43, 524.62) | 4.54 (-6.36, 14.59) |  |
| Spain | | 17.19 (18.23, 15.58) | 7.99 (8.75, 6.86) | -53.49 (-57.3, -49.45) | 606.01 (743.5, 490.72) | | 553.13 (741.35, 398.04) | -8.73 (-19.76, 2.11) |  |
| Sweden | | 8.42 (9.02, 7.64) | 7.93 (8.57, 7.03) | -5.83 (-13.16, 1.77) | 328.54 (397.89, 266.85) | | 408.01 (518.15, 313.64) | 24.19 (14.26, 33.49) |  |
| Switzerland | | 13.42 (14.27, 12.18) | 6.42 (7.04, 5.51) | -52.19 (-56.21, -48.13) | 431.55 (528.88, 355.38) | | 405.89 (546.32, 300.49) | -5.95 (-17.02, 4.64) |  |
| United Kingdom | | 7.9 (8.19, 7.35) | 4.29 (4.52, 3.88) | -45.76 (-48.09, -43.57) | 370.21 (461.16, 295.31) | | 589.17 (787.81, 419.71) | 59.14 (41.29, 73.76) |  |
| Monaco | | 3.03 (3.71, 2.43) | 3.1 (3.68, 2.51) | 2.1 (-20.73, 31.98) | 205.13 (269.22, 151.54) | | 350.06 (472.45, 247.12) | 70.65 (54.97, 85.64) |  |
| San Marino | | 7.05 (8.33, 5.91) | 5.79 (7.99, 3.93) | -17.86 (-46.16, 16.94) | 288.47 (367.46, 224.02) | | 415.16 (550.86, 302.95) | 43.92 (24.38, 62.06) |  |

## Table S8 All-age high fasting plasma glucose DALYs due to specific causes in 2019 by the Europe regions.

| location | Cardiovascular diseases | Chronic kidney disease | Neoplasms | Neurological disorders | Sense organ diseases | Tuberculosis | Diabetes mellitus type 1 | Diabetes mellitus type 2 |
| --- | --- | --- | --- | --- | --- | --- | --- | --- |
|  | Rate (95% UI) | Rate (95% UI) | Rate (95% UI) | Rate (95% UI) | Rate (95% UI) | Rate (95% UI) | Rate (95% UI) | Rate (95% UI) |
| Global | 938.2 (685.9, 1283.7) | 169.2 (143.5, 196.6) | 110.9 (30.5, 227.1) | 31.6 (5.4,101.7) | 8.7 (2.1, 20.2) | 49.1 (31.2, 67.2) | 59.2 (50.5, 69.6) | 856.9 (717.0, 1021.1) |
| Europe | 1439.9 (1007.9, 2029.7) | 98.0 (78.5, 119.7) | 248.2 (69.5, 70.0) | 70.0 (12.0, 224.8) | 6.6 (1.7, 15.0) | 6.7 (4.2, 9.2) | 67.8 (52.4, 87.5) | 927.2 (722.9,1156.4) |
| Eastern Europe | 2100.5 (1510.8, 2936.1) | 55.5 (42.9, 69.5) | 125.6 (32.9, 266.5) | 33.3 (5.2, 109.1) | 3.4 (0.7, 7.9) | 16.3 (9.8, 23.2) | 61.4 (48.1, 77.8) | 597.2 (471.3,744.0) |
| Central Europe | 2205.7 (1502.1, 3129.3) | 102.8 (79.2, 128.60) | 368.6 (101.1, 769.0) | 75.8 (13.4,242.8) | 4.6 (1.1, 10.5) | 7.1 (4.4, 9.8) | 74.9 (60.7, 92.3) | 1295.1 (1004.2,1623.7) |
| Western Europe | 976.9 (644.6, 1451.4) | 97.8 (76.9, 123.5) | 296.4 (84.6, 592.7) | 92.5 (16. 0,300.0) | 8.3 (2.1, 18.9) | 1.8 (1.1, 2.7) | 68.8 (50.2, 93.1) | 973.8 (748.9, 1238.5) |

## Table S9 The Socio-Demographic Indices (SDI) values from 1990 to 2019, by the Europe regions

| Location | 1990 | 1991 | 1992 | 1993 | 1994 | 1995 | 1996 | 1997 | 1998 | 1999 | 2000 | 2001 | 2002 | 2003 | 2004 | 2005 |
| --- | --- | --- | --- | --- | --- | --- | --- | --- | --- | --- | --- | --- | --- | --- | --- | --- |
| Eastern Europe | 0.68 | 0.687 | 0.697 | 0.702 | 0.702 | 0.705 | 0.707 | 0.708 | 0.709 | 0.711 | 0.711 | 0.713 | 0.716 | 0.72 | 0.727 | 0.734 |
| Central Europe | 0.641 | 0.647 | 0.652 | 0.658 | 0.665 | 0.672 | 0.678 | 0.683 | 0.689 | 0.695 | 0.702 | 0.709 | 0.715 | 0.72 | 0.726 | 0.731 |
| Western Europe | 0.75 | 0.756 | 0.762 | 0.767 | 0.772 | 0.775 | 0.779 | 0.782 | 0.784 | 0.787 | 0.79 | 0.794 | 0.797 | 0.8 | 0.802 | 0.805 |
| **Attached with Supplementary Table 5** | | | | | | | | | | | | | | | | |
| Location | 2005 | 2006 | 2007 | 2008 | 2009 | 2010 | 2011 | 2012 | 2013 | 2014 | 2015 | 2016 | 2017 | 2018 | 2019 | 2006 |
| Eastern Europe | 0.734 | 0.74 | 0.745 | 0.751 | 0.757 | 0.762 | 0.765 | 0.768 | 0.772 | 0.777 | 0.781 | 0.785 | 0.788 | 0.791 | 0.793 | 0.74 |
| Central Europe | 0.731 | 0.736 | 0.74 | 0.745 | 0.75 | 0.756 | 0.76 | 0.764 | 0.768 | 0.771 | 0.775 | 0.778 | 0.781 | 0.785 | 0.788 | 0.736 |
| Western Europe | 0.805 | 0.807 | 0.81 | 0.812 | 0.815 | 0.817 | 0.821 | 0.824 | 0.827 | 0.83 | 0.832 | 0.835 | 0.838 | 0.841 | 0.843 | 0.807 |
